# Multistate Redox Switching and Near-Infrared Electrochromism Based on a Star-Shaped Triruthenium Complex with a Triarylamine Core

**DOI:** 10.1038/srep35253

**Published:** 2016-10-12

**Authors:** Jian-Hong Tang, Yan-Qin He, Jiang-Yang Shao, Zhong-Liang Gong, Yu-Wu Zhong

**Affiliations:** 1 Beijing National Laboratory for Molecular Sciences, CAS Key Laboratory of Photochemistry, Institute of Chemistry, Chinese Academy of Sciences, Beijing 100190, China; 2 University of Chinese Academy of Sciences, Beijing 100049, China

## Abstract

A star-shaped cyclometalated triruthenium complex **2**(PF_6_)_n_ (n = 3 and 4) with a triarylamine core was synthesized, which functions as a molecular switch with five well-separated redox states in both solution and film states. The single-crystal X-ray structure of **2**(PF_6_)_3_ is presented. This complex displays four consecutive one-electron redox waves at +0.082, +0.31, +0.74, and +1.07 V vs Ag/AgCl. In each redox state, it shows significantly different NIR absorptions with *λ*_max_ of 1590 nm for **2**^4+^, 1400 nm for **2**^5+^, 1060 nm for **2**^6+^, and 740 nm for **2**^7+^, respectively. Complex **2**^4+^ shows a single-line EPR signal at *g* = 2.060, while other redox states are all EPR inactive. The spin density distributions and NIR absorptions in different redox states were rationalized by DFT and TDDFT calculations. A vinyl-substituted triruthenium analogous **3**(PF_6_)_4_ was prepared, which was successfully polymerized on ITO glass electrode surfaces by reductive electropolymerization. The obtained poly-**3**^n+^/ITO film was characterized by FTIR, AFM, and SEM analysis. It shows four well-defined redox couples and reversible multistate NIR electrochromism. In particular, a contrast ratio (ΔT%) up to 63% was achieved at the optic telecommunication wavelength (1550 nm).

Molecular switches can be reversibly shifted between two or more stable states in response to external stimuli^1^. Among them, electro-active compounds have received much interest as redox-driven molecular switches^2^, which show appealing functions such as information storage^3^,^4^,^5^, electrochromism^6^,^7^,^8^, electrofluorochromism^9^, and switching of molecular conductance^10^ and magnetism^11^.

Recent attention has been focusing on the design of smart and sophisticated switching molecules that can display multiple and consecutive redox processes at low potentials with each redox state being distinguished by specific readout signals^12^,^13^,^14^. This is particularly important for the application of these materials in information storage because the information density can be significantly increased in the form of multistate memory^15^,^16^. Redox switches with two or three states are very common^3^,^4^,^5^,^6^,^7^,^8^,^9^,^10^. However, the realization of four or more than four redox states is challenging^12^,^13^,^14^,^15^,^16^. In order to get multistate redox switches, molecules should display multistate redox processes at a relatively low region. Otherwise, the higher or lower redox states will be quickly reduced or oxidized by the atmosphere or solvent. In addition, the potential separation between neighboring states should be large to prevent disproportionation. A third consideration is that known molecular switches are mostly based on solution state. For eventual applications, these solution-based demonstrations must be transformed into surface-confined technologies^17^,^18^,^19^,^20^. It is thus critical to take into account of a proper film-formation method during the stage of molecular design.

Recent studies have shown that polypyridyl ruthenium complexes with a Ru-C bond, namely cyclometalated ruthenium complexes, are particularly useful for the construction of molecular switches with multiple redox processes^21^,^22^. The presence of the Ru-C bond significantly decreases the Ru(III/II) potential and affords diruthenium^23^ or ruthenium-amine^24^,^25^,^26^ conjugated complexes with three redox states. These complexes are characterized by rich absorptions in the visible and near-infrared (NIR) region and potentially useful as electrochromic materials for variable optical attenuators in fiber-optic communications^27^,^28^,^29^. The film formation of these complexes can be realized by electropolymerization^30^, layer-by-layer assembly^31^, and self-assembly monolayers^32^. Recently, a cyclometalated diruthenium system bridged by a redox-active amine unit was reported by us^33^,^34^, which showed the presence of four redox states at a low potential region. We present in this contribution the synthesis and study of a star-shaped tris-cyclometalated ruthenium complex with an amine core. The synthesis is very simple and straightforward. This complex functions as a molecule switch with readily available five redox states at a low potential window. In addition, electropolymerized films of a vinyl-functionalized derivative were prepared, which were used to examine the redox switching behavior in film state.

## Results

**Figure 1:**
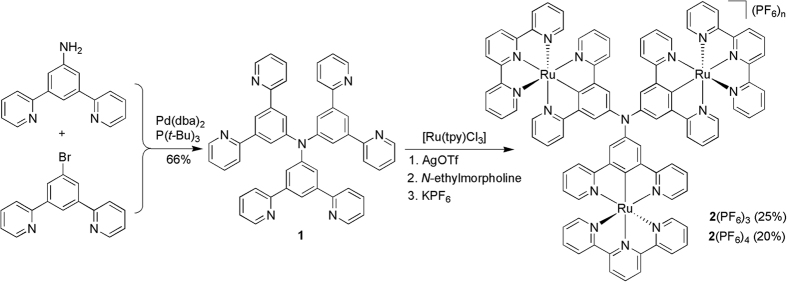
Synthesis of **2**(PF_6_)_3_ and **2**(PF_6_)_4_.

As depicted in Fig. 1, ligand **1** was synthesized through the palladium-catalyzed C-N couplings between 3,5-di(pyrid-2-yl)bromobenzene and 3,5-bis(pyrid-2-yl)aniline. The product precipitated out from the reaction mixture when the reaction was complete. After cooling to room temperature, a simple filtration and washing procedure gave ligand **1** in 66% yield, which is pure enough for the next transformation without further purification. The reaction of [Ru(tpy)Cl_3_] (tpy = 2,2′:6′,2″-terpyridine) with **1** under microwave heating, in the presence of AgOTf and *N*-ethylmorpholine and followed by anion exchange using KPF_6_, afforded complexes **2**(PF_6_)_3_ and **2**(PF_6_)_4_, in a total yield of 45%. Complex **2**(PF_6_)_4_ with four counter anions was the one-electron-oxidized form of **2**(PF_6_)_3_. Satisfactory ^1^H NMR spectrum could be obtained for **2**(PF_6_)_3_ in the presence of small amount of aqueous hydrazine in CD_3_CN, while **2**(PF_6_)_4_ is paramagnetic and no distinct NMR data was obtained. Both compounds are bench stable and satisfactory microanalysis was obtained. The observed isotope distribution is in agreement with the theoretical value (See details in the Supporting Information).

**Figure 2:**
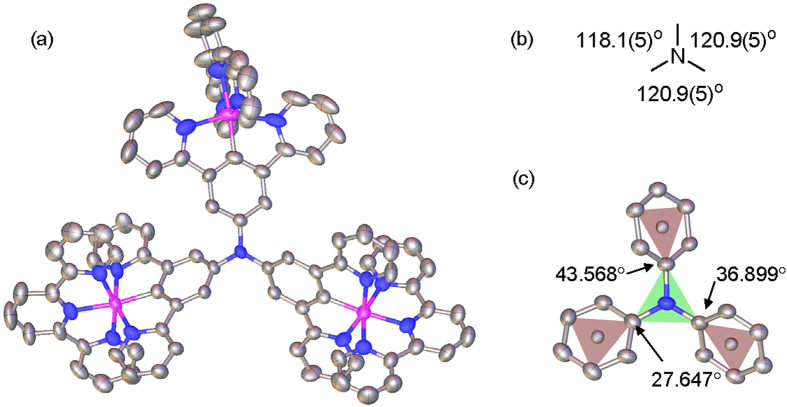
(**a**) Thermal ellipsoid plot at 30% probability of the single-crystal X-ray structure of **2**^3+^. H atoms are omitted. Color code: carbon, grey; nitrogen, blue; ruthenium, magenta. (**b**,**c**) Schematic representation showing the degrees of the three ∠CNC angle around the central amine atom and the dihedral angles between the central amine plane and each cyclometalating phenyl ring.

Partially due to the large size of the molecule, we failed to obtain a single crystal of **2**(PF_6_)_3_. Fortunately, a single crystal of **2**(BPh_4_)_3_, which was produced by the anion exchange of **2**(PF_6_)_3_ with NaBPh_4_, was obtained suitable for X-ray diffraction analysis (Fig. 2). The molecule is pesudo-*C*_3_ symmetric around the central amine, which has essentially a planar configuration. The three ∠CNC angle around the central amine atom is around 120° and the triarylamine unit has a three-wheel propeller configuration. The degrees of the dihedral angles between the central amine plane and each cyclometalating phenyl ring are 27.647°, 36.899°, and 43.568°, respectively. Each ruthenium component has an expected octahedral six-coordinate configuration. The terminal tpy ligand is orthogonal to the N^∧^C^∧^N cyclometalating ligand plane of the same ruthenium component. The three Ru-C bonds are 1.941(7), 1.942(6), and 1.953(6) Å in length, respectively. The Ru-N bonds around the ruthenium ion are in the range of 1.99–2.10 Å in length. The distance between each ruthenium ion and the central amine nitrogen atom is around 6.15 Å. Attempts to get a high-quality single crystal of **3**(PF_6_)_4_ or **3**(BPh_4_)_4_ failed at this stage.

**Figure 3:**
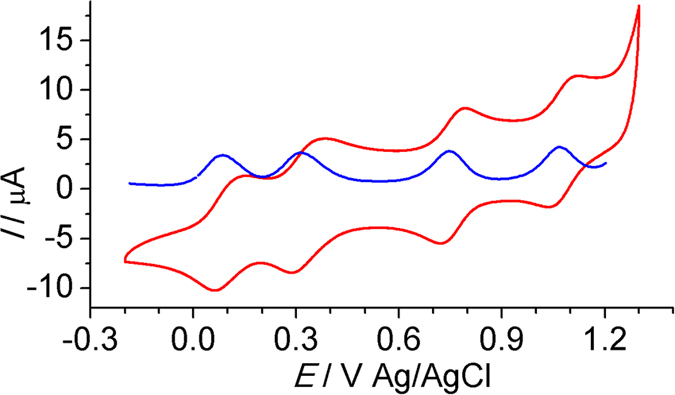
Anodic CV (red curve) and DPV (blue curve) of **2**(PF_6_)_3_ in 0.1 M Bu_4_NClO_4_/CH_3_CN.

Figure 3 shows the anodic cyclic voltammogram (CV) and differential pulse voltammogram (DPV) of **2**(PF_6_)_3_ in CH_3_CN. Four consecutive one-electron waves at +0.082, +0.31, +0.74, and +1.07 V vs Ag/AgCl are observed (annotated as *E*_1_–*E*_4_, respectively). The peak-to-peak potential difference of the oxidation and reduction of each couple is around 60~70 mV. These waves are ascribed to the stepwise oxidations of three cyclometalated ruthenium components and the central amine unit. Notable is that these four redox couples all locate at a relatively low potential region and are well-separated from each other. The potential separation between neighboring redox waves is 230, 430, and 300 mV for Δ*E*_1−2_, Δ*E*_2−3_, and Δ*E*_3−4_, respectively. This ensures a high thermodynamic stability of each redox state against proportionation. The comproportionation was calculated to be 7.9 × 10^3^, 1.9 × 10^7^, 1.2 × 10^5^, respectively, by *K*_c_ = 10^Δ*E*/59^. At a further positive potential, multi-electron redox waves at +1.31 V and irreversible oxidation peaks at +1.58 V are observed (Figure S1). The previously reported amine-bridged diruthenium complex show three consecutive anodic waves at +0.21, +0.44, and +1.03 V, respectively^33^. This indicates that the attachment of the third ruthenium component can further decrease the *E*_1_ potential by more than 100 mV, which is important for maintaining the high stability of **2**^3+^ and **2**^4+^. The first cathodic wave of **2**(PF_6_)_3_ occurs at −1.25 V, followed by adsorption/desprotion peaks at a further negative potential (Figure S1). These waves have relatively high redox potentials and are not considered for the applications as molecular switches.

**Figure 4:**
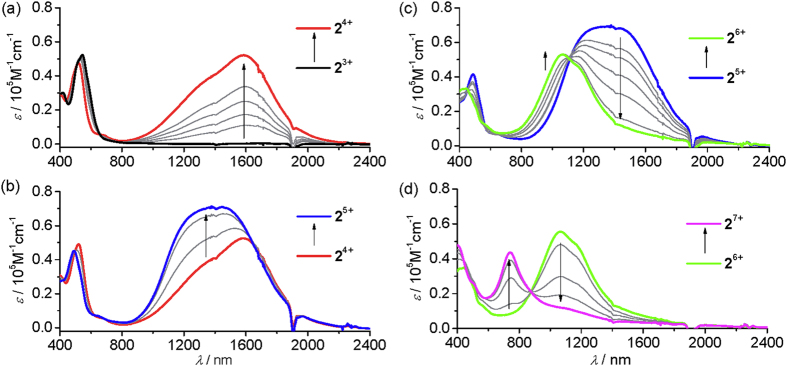
Absorption spectral changes of **2**(PF_6_)_3_ upon stepwise oxidation with cerium ammonium nitrate in CH_3_CN.

As was discussed above, complex **2**(PF_6_)_3_ shows four consecutive redox waves at a low potential region with large potential separation between neighboring waves. This feature makes complex **2**(PF_6_)_3_ an appealing redox switch. Figure 4 shows the absorption spectral changes of **2**(PF_6_)_3_ in CH_3_CN during the one-electron (single-), the second one-electron (double-), the third one-electron (triple-), and the forth one-electron (quadruple) oxidation with up to one, two, three, and four equiv of cerium ammonium nitrate (CAN), respectively. The one-electron-oxidized state **2**^4+^ was characterized by an intense NIR absorption band at 1590 nm. The previously isolated sample **2**(PF_6_)_4_ shows exactly the same NIR absorption. Upon transforming into higher-oxidation states, the NIR absorptions of these complexes moved to 1400 nm for **2**^5+^, 1060 nm for **2**^6+^, and 740 nm for **2**^7+^, respectively. The above four step spectral changes basically can be reproduced by spectroelectrochemical measurements at a transparent indium-tin-oxide (ITO) glass electrode (Figure S2). The potentials applied for each step electrolysis correlates well with the redox processes shown in Fig. 3. These spectral changes are reversible when an opposite potential was applied, suggesting the good stability of each redox state during the redox switching process.

**Figure 5:**
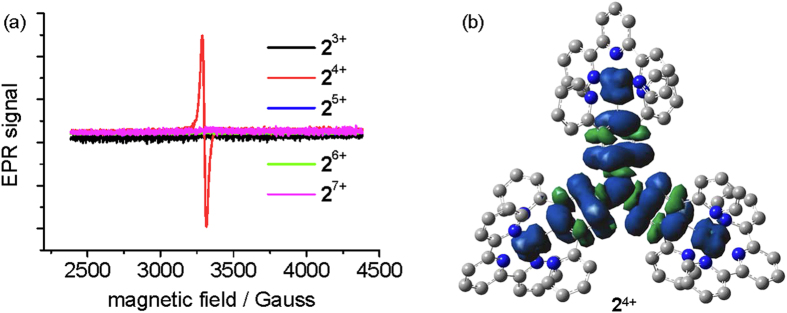
(**a**) EPR signals of **2**^n+^ (n = 3–7) at 77 K in frozen CH_3_CN. (**b**) Spin density distribution of **2**^4+^ calculated at the level of UCAM-B3LYP/LANL2DZ/6-31G*/CPCM.

In addition to the NIR absorption spectra, the different redox states of **2** can be distinguished by the EPR signal. Complex **2**^4+^ shows an intense single-line EPR signal at *g* = 2.060 (Fig. 5a) at either rt or 77 K in frozen CH_3_CN. However, other redox states (**2**^3+^, **2**^5+^, **2**^6+^, and **2**^7+^) are all EPR inactive. Complexes with a high oxidation state were obtained from **2**(PF_6_)_3_ upon oxidation with CAN and the identities were confirmed by the NIR absorption spectra. The single-line EPR signal of **2**^4+^ suggests that it has largely an amine-associated free spin supported by three ruthenium units. A low-spin Ru(III) complex often displays an axial or rhombic EPR signal at low temperature^35^. Note that **2**(PF_6_)_4_ is a highly stable paramagnetic amminium radical cation, and no changes occur to its NIR absorption spectrum and EPR signal after storing at ambient conditions after several months in the solid state. The topic of obtaining stable paramagnetic organic radicals have recently received intense interest due to their unique properties^36^,^37^,^38^,^39^.

The absorptions of **2**^7+^ at 740 nm are easy to understand. It is assigned to the N^•+^-localized transitions of triarylaminium compounds^40^,^41^. DFT calculations were performed on **2**^4+^ using UCAM-B3LYP/LANL2DZ/6-31G*/CPCM. The spin density of **2**^4+^ is distributed across the N(phenyl-Ru)_3_ backbone (Fig. 5b). The central amine nitrogen atom has a Mulliken spin density of 0.331, while the contribution of each ruthenium ion is 0.070, 0.071, and 0.074, respectively. Three cyclometalating phenyl rings have contributions of 0.154, 0.156, and 0.160, respectively. This suggests that the interpretation of **2**^4+^ as a ruthenium-supported triphenylaminium radical cation is appropriate. The use of B3LYP produced a more delocalized spin density distribution (Figure S3). TDDFT calculations of **2**^4+^ show that the NIR transitions are largely associated with the *β* spin transitions from the ruthenium-dominated highest occupied spin orbital (HOSO) and HOSO-1 to the lowest unoccupied spin orbital (LUSO) delocalized over the N(phenyl-Ru)_3_ backbone (Figure S4). The NIR absorptions of **2**^5+^ and **2**^6+^ could also be reproduced by TDDFT calculations (Figures S5 and S6).

The redox switching shown in Fig. 4 was performed in solution. To demonstrate the reversibility and practicality of this switching behavior, it would be necessary to obtain thin films of this complex in good quality. The relatively low solubility of the trimetallic complex in common organic solvents make the film formation by conventional spin coating unsuitable. The reductive electropolymerization method for obtaining thin films was thus used^42^,^43^,^44^.

**Figure 6:**
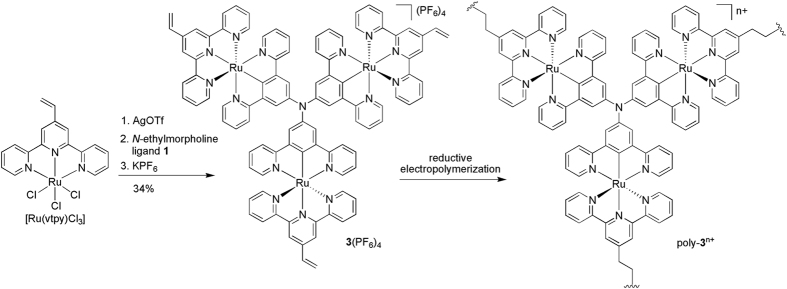
Synthesis of complex **3**(PF_6_)_4_ and the schematic representation of the reductive electropolymerization of **3**(PF_6_)_4_.

**Figure 7:**
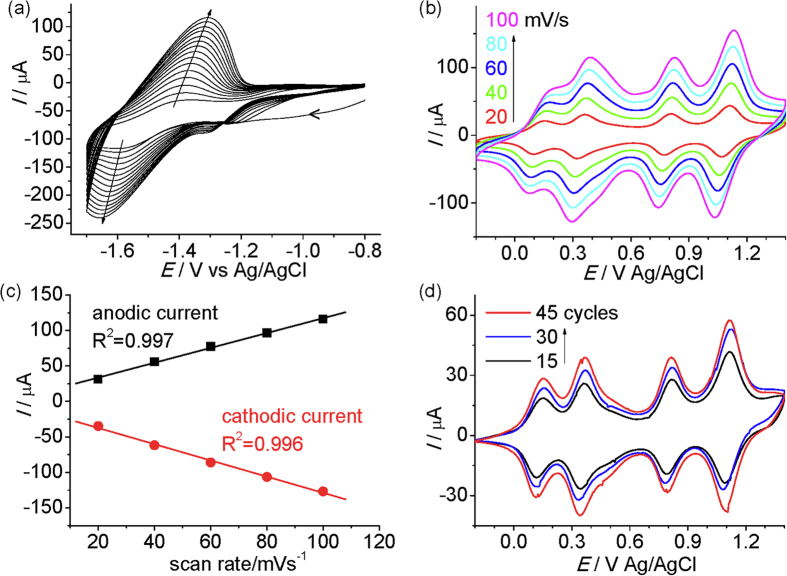
(**a**) CVs recorded during the reductive electropolymerization of **3**(PF_6_)_4_ (around 0.5 mM in CH_3_CN) at an ITO glass electrode by 15 potential cycles between −0.8 and −1.7 V). (**b**) CVs of the poly-**3**^n+^/ITO film obtained in (**a**) at different scan rates (20, 40, 60, 80, and 100 mV/s, respectively). (**c**) Linear dependence of the peak currents of the redox wave at +0.36 V in (**b**) as a function of scan rate. (**d**) CVs of poly-**3**^n+^/ITO films obtained after 15, 30, and 45 potential cycles. The scan rate is 20 mV/s. The surface coverage is 3.0 × 10^−9^, 5.0 × 10^−9^, and 8.0 × 10^−9^ mol/cm^2^, respectively.

Complex **3**(PF_6_)_4_ with a vinyl substituent on each terminal ligand was prepared from [Ru(vtpy)Cl_3_] (vtpy = 4′-vinyl-2,2′:6′,2″-terpyridine)^45^ and the C_3_-symmetric bridging ligand **1** (Fig. 6). The reductive electropolymerization of **3**(PF_6_)_4_ was smoothly executed on ITO glass electrode surface via repeatedly cathodic potential sweeps between −0.8 V and −1.7 V in CH_3_CN, as evidenced by the gradual and continuous increase in current (Fig. 7a). The obtained poly-**3**^n+^/ITO film shows similar four well-defined redox processes during the anodic sweep in clear electrolyte solution as have been observed for **2**(PF_6_)_4_ in solution, and the peak currents are linearly dependent on the scan rate (Fig. 7b,c). The latter feature is characteristic of redox processes confined on electrode surfaces. The peak-to-peak potential separation of each redox wave is around 30 mV at a low scan rate of 20 mV/s. It increases to around 100 mV at a high scan rate of 100 mV/s. The surface coverage can be estimated by the integration of the charge under each redox wave. The thickness or surface coverage of the film can be easily controlled by changing the potential cycles during polymerization (Fig. 7d).

The FTIR spectra of the monomer complex **3**(PF_6_)_4_ and the poly-**3**^n+^ sample are displayed in Figure S7. The intense peak at 840 cm^−1^ of **3**(PF_6_)_4_ is attributed to the PF_6_^−^ stretches. The signal around 1100 cm^−1^ of poly-**3**^n+^ is assigned to the ClO_4_^−^ anions. This indicates that the anions of the obtained poly-**3**^n+^ samples are mainly ClO_4_^−^, which was incorporated from the electrolyte (Bu_4_NClO_4_) during electropolymerization. The weak peak at 960 cm^−1^ of **3**(PF_6_)_4_ is caused by the out-of-plane deformation vibration of vinyl groups^46^. This peak is essentially absent in the polymer sample, which suggests that all of the three vinyl groups of the monomer are consumed during the polymerization.

**Figure 8:**
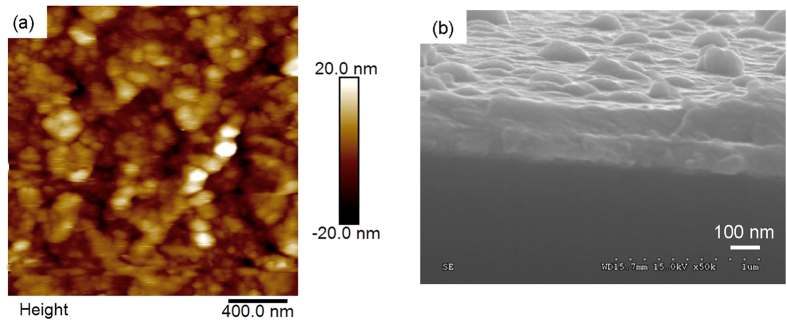
AFM height image (**a**) (2 × 2 μm) and SEM cross section image of poly–**3**^n+^**/**ITO film with *Γ* of 8.0 × 10^−9^ mol/cm^2^.

Figure 8a shows the typical AFM surface morphology of the poly-**3**^n+^/ITO film. The size of islands varies from a few tens of nm to a hundred nm. The surface has a mean roughness (rms) of 6.03 nm. As measured by a scratching method (Figure S8), the film with a surface coverage of 8.0 × 10^–9 ^mol/cm^2^ is around 100 nm thick. The cross-section SEM image of the film confirms a similar thickness (Fig. 8b). In addition, the SEM image suggests that the film adheres tightly to the ITO glass, which is critical for the performance of redox switching in the film state.

**Figure 9:**
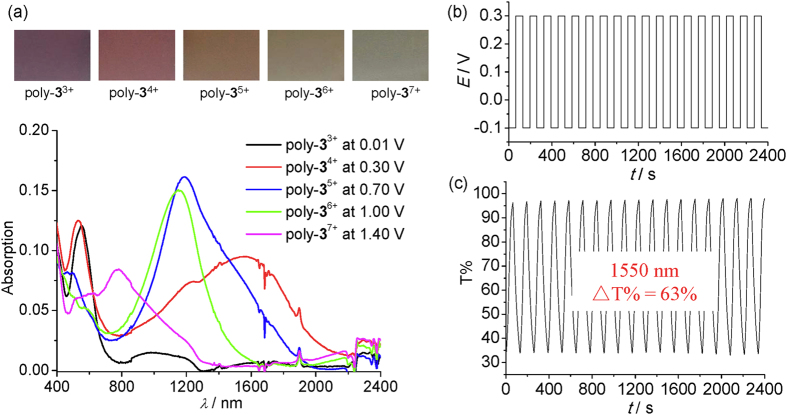
(**a**) Absorption spectra and film picture of the poly–**3**^n+^**/**ITO film at different redox states (*Γ* = 3.0 × 10^−9^ mol/cm^2^). (**b**) Current assumption and (**c**) transmittance changes monitored at λ = 1550 nm as a function of time during the electrochromic switching of the poly–**3**^n+^**/**ITO film (*Γ* = 2.0 × 10^−8^ mol/cm^2^) between −0.1 and +0.3 V in 0.1 M Bu_4_NClO_4_/CH_3_CN. The interval is 65 s.

The poly-**3**^n+^/ITO film shows four-step redox switching as have been observed for **2**(PF_6_)_4_ in solution (Figure S9). The absorption spectra and the film picture of the poly-**3**^n+^/ITO film at different redox states are shown in Fig. 9a. The film is purple, brown, chocolate, olive, and blue from poly-**3**^3+^ through poly-**3**^7+^, respectively. The absorption spectra in the NIR region of the five redox states of the film are significantly different. However, poly-**3**^3+^ and poly-**3**^4+^ display very absorption spectra in the visible region. Similar situation is observed for poly-**3**^5+^ and poly-**3**^6+^. This explains why the film color does not change significantly among these redox states. The spectral changes recorded during the electrochemical switching are fully reversible. In particular, the film displays a high contrast ratio (ΔT%) of 63% at the fiber communication wavelength (1550 nm) during the double-potential-step chronoamperometry measurements between −0.1 and +0.3 V (Fig. 9b,c), which is much higher with respect to that of the electrochromism based on a recently reported diruthenium complex (ΔT%: around 30∼40%)^33^. To the best of our knowledge, this is also one of the highest contrast ratios ever achieved at the optic telecommunication wavelength on the basis of molecular electrochromic materials^29^,^47^,^48^,^49^,^50^. The coloration efficiency (CE) of for the above process was calculated to be 250 cm^2^/C according to the equation CE(λ) = ΔOD/Q_d_, where ΔOD = log[T_*b*_/T_*c*_], OD is optical density, Q_d_ is the injected/ejected charge density (C/cm^2^), and T_*b*_ and T_*c*_ are the transmittance in the bleached and colored states at 1550 nm. The response time for the contrast ratio to reach over 90% of its maximum is around 50 s (Figure S10). This response time is much longer with respect to the NIR electrochromism based on our previously reported diruthenium complexes (around 5 s)^21^,^33^. One possible reason is that the currently used triruthenium monomer contains three vinyl groups and the resulting poly-**3**^n+^ film has a higher degree of cross-linking and chain entanglement relative to the previously reported diruthenium monomers with two vinyl groups. In this sense, the anion transport to compensate the charge changes of the polymer backbones of the triruthenium film will be severely hindered, leading to a long response time.

In addition to the first-step redox switching, the electrochromism of the poly-**3**^n+^/ITO film at 1180 nm switched between −0.1 and +0.7 V (the second oxidation), 1150 nm switched between −0.1 and +1.0 V (the third oxidation), and 780 nm switched between −0.1 and +1.4 V (the forth oxidation) were examined (Figure S11). The second and third oxidation step show good switching cycling reversibility, with a contrast ratio of around 60% being achieved for both cases. However, reversibility of the forth oxidation step is poor. After 25 cycles of redox switching between −0.1 and +1.4 V, the contrast ratio decreased from 42% to 30% (Figure S11c). In addition, the optical memory of the different redox states of the poly-**3**^n+^/ITO film was examined (Figure S12). The poly-**3**^3+^ and poly-**3**^4+^ are two bench-stable states. The poly-**3**^5+^ and poly-**3**^6+^ states also have long optical memory time. For instance, when the polymer film was oxidized to poly-**3**^5+^ by a potential at +0.7 V, the transmittance at 1180 nm changed slightly from 40% to 55% after 100 min after the potential was switched off. Similar situation is observed for the poly-**3**^6+^ state. However, the optical stability of poly-**3**^7+^ is rather poor. The optical memory time of poly-**3**^7+^ is only around 5 min. The high oxidation state of **3**^7+^ makes it easily reduced by solvents or atmosphere.

In summary, we have successfully prepared a star-shaped tris-cyclometalated ruthenium complex with a redox-active triarylamine core. Interestingly, the one-electron-oxidized form was isolated as a stable paramagnetic compound, which is best described as a ruthenium-stabilized triphenylaminium radical cation as supported by EPR and DFT analysis. This complex and the electropolymerized films of the vinyl-functionalized complex display four consecutive one-electron anodic redox couples at low potentials, functioning as a molecular redox switch with up to five well-separated states. NIR absorptions and EPR signal were used as the output signals to distinguish different redox states. This makes the complex attractive for high-density information storage^15^,^16^. In addition, a high contrast ratio of 63% was achieved at the optic telecommunication wavelength (1550 nm). This is one of the highest contrast ratios ever achieved at this wavelength on the basis of molecular electrochromic materials, and it is potentially useful as active materials for variable optical attenuators in optic telecommunication^29^,^47^,^48^,^49^,^50^.

## Methods

### Spectroscopic Measurements

Absorption spectra were recorded using a PE Lambda 750 UV/vis/NIR spectrophotometer at room temperature. Spectroelectrochemical measurements and electrochromic studies were performed in a thin layer cell (optical length = 0.2 cm), in which an ITO glass electrode (<10 Ω/square) or the ploy-**3**^n+^/ITO film was set as the working electrode in 0.1 M Bu_4_NClO_4_/CH_3_CN. A platinum wire and Ag/AgCl in saturated aqueous NaCl solution was used as the counter electrode and reference electrode, respectively. The cell was put into the spectrophotometer to monitor spectral changes during electrolysis.

### Electrochemical Measurements

Electrochemical measurements were taken using a CHI 660D potentiostat with one-compartment electrochemical cell under an atmosphere of nitrogen. All measurements were carried out in 0.1 M Bu_4_NClO_4_ in CH_3_CN. The working electrode was a glassy carbon with a diameter of 3 mm. The electrode was polished prior to use with 0.05 μm alumina and rinsed thoroughly with water and acetone. A large area platinum wire was used as the counter electrode. All potentials are referenced to a Ag/AgCl electrode in saturated aqueous NaCl without compensation for the liquid junction potential. Electropolymerization experiments were carried out in three compartment electrochemical cell with ITO glass (<10 Ω/square) as the working electrode. The ITO glass was cleaned by successive sonication in deionized water, isopropanol and acetone for 10 min, followed by drying with N_2_ gas. The obtained film has a typical size of 1.0 cm × 0.8 cm.

### X-ray Crystallography

The X-ray diffraction data were collected using a Rigaku Saturn 724 diffractometer on a rotating anode (Mo-K radiation, 0.71073 Å) at 173 K. The structure was solved by the direct method using SHELXS-97 and refined with Olex2. Crystallographic data for **2**(BPh_4_)_3_·CH_2_Cl_2_ (CCDC 1480175): C_93_H_63_N_16_Ru_3_·3(BC_24_H_20_)·CH_2_Cl_2_, *M* = 2750.35, monoclinic, space group P 1 21/n 1, *a* = 19.981(4), *b* = 31.846(5), *c* = 26.234(5) Ǻ, *α* = 90°, *β* = 107.802(2)°, *γ* = 90°, *U* = 15894(5) Ǻ^3^, T = 173 K, *Z* = 4, 130185 reflections measured, radiation type MoK\*a*, radiation wavelength 0.71073 Ǻ, final R indices R1 = 0.1226, wR2 = 0.2660, R indices (all data) R1 = 0.1348, wR2 = 0.2734.

### EPR Measurements

EPR measurements were performed on a Bruker ELEXSYS E500-10/12 spectrometer at 77 K in frozen CH_3_CN. The spectrometer frequency is 9.7 × 10^9^ Hz.

### AFM Images

AFM images were taken using a Nanoscope III A multimode atomic force microscope (Vecco Inc., USA) in the ScanAsyst mode in air with a scan speed of 1 Hz.

### SEM Images

SEM images were taken using a JEOL S-4300 field emission scanning microscope operated at an acceleration voltage of 15 kV. Prior to measurement, an ultrathin conductive Au coating was deposited on the top of the polymeric films on ITO glass electrodes.

## Additional Information

**How to cite this article**: Tang, J.-H. *et al*. Multistate Redox Switching and Near-Infrared Electrochromism Based on a Star-Shaped Triruthenium Complex with a Triarylamine Core. *Sci. Rep*. **6**, 35253; doi: 10.1038/srep35253 (2016).

## Supplementary Material

Supplementary Information
