# Supplementary material for: Multistate Redox Switching and Near-Infrared Electrochromism Based on a Star-Shaped Triruthenium Complex with a Triarylamine Core
Source: Sci Rep. 2016 Oct 12;6:35253. doi: 10.1038/srep35253 (PMC5059756; doi:10.1038/srep35253)
Supplement: Supplementary Information [file srep35253-s1.pdf]

Supporting Information for:

Multistate Redox Switching and Near-Infrared Electrochromism Based on a Star-Shaped Triruthenium Complex with a Triarylamine Core

Jian-Hong Tang,[a,b] *Yan-Qin He,*[a] *Jiang-Yang Shao,*[a] *Zhong-Liang Gong,*[a] *and Yu-Wu Zhong**,[a,b]

[a]Beijing National Laboratory for Molecular Sciences, CAS Key Laboratory of Photochemistry, Institute of Chemistry, Chinese Academy of Sciences, Beijing 100190, China.

[b]University of Chinese Academy of Sciences, Beijing 100049, China.

*Email: zhongyuwu@iccas.ac.cn

Electrochemical Measurements. Electrochemical measurements were taken using a CHI 660D potentiostat with one-compartment electrochemical cell under an atmosphere of nitrogen. All measurements were carried out in 0.1 M Bu_4NClO_4 in CH_3CN. The working electrode was a glassy carbon with a diameter of 3 mm. The electrode was polished prior to use with 0.05 μm alumina and rinsed thoroughly with water and acetone. A large area platinum wire coil was used as the counter electrode. All potentials are referenced to a Ag/AgCl electrode in saturated aqueous NaCl without compensation for the liquid junction potential. Electropolymerization experiments were carried out in three compartment electrochemical cell with ITO glass (< 10 Ω/square) as the working electrode. The ITO glass was cleaned by successive sonication in deionized water, isopropanol and acetone for 10 min, followed by drying with N_2 gas. The obtained film has a typical size of 1.0 cm × 0.8 cm.

Spectroscopic Measurements. Absorption spectra were recorded using a PE Lambda 750 UV/vis/NIR spectrophotometer at room temperature. Spectroelectrochemical measurements and electrochromic studies were performed in a thin layer cell (optical length = 0.2 cm), in which an ITO glass electrode (< 10 Ω/square) or the ploy-**3**$^{n+}$/ITO film was set as the working electrode in 0.1 M Bu_4NClO_4/CH_3CN. A platinum wire and Ag/AgCl in saturated aqueous NaCl solution was used as the counter electrode and reference electrode, respectively. The cell was put into the spectrophotometer to monitor spectral changes during electrolysis.

AFM Images. AFM images were taken using a Nanoscope III A multimode atomic force microscope (Vecco Inc., USA) in the ScanAsyst mode in air with a scan speed of 1 Hz.

SEM Images. SEM images were taken using a JEOL S-4300 field emission scanning microscope operated at an acceleration voltage of 15 kV. Prior to measurement, an ultrathin conductive Au coating was deposited on the top of the polymeric films on ITO glass electrodes.

EPR Measurements. EPR measurements were performed on a Bruker ELEXSYS E500-10/12 spectrometer at rt or 77 K in frozen CH_3CN. The spectrometer frequency is 9.7×10^9 Hz.

X-ray Crystallography. The X-ray diffraction data were collected using a Rigaku Saturn 724 diffractometer on a rotating anode (Mo-K radiation, 0.71073 Å) at 173 K. The structure was solved by the direct method using SHELXS-97[1] and refined with Olex2.[2]

Crystallographic data for **2**$(BPh_4)_3 \cdot CH_2Cl_2$ (CCDC 1480175): $C_{93}H_{63}N_{16}Ru_3 \cdot 3(BC_{24}H_{20}) \cdot CH_2Cl_2$, M = 2750.35, monoclinic, space group P 1 21/n 1, a = 19.981(4), b = 31.846(5), c = 26.234(5) Å, α = 90°, β = 107.802(2)°, γ = 90°, U = 15894(5) Å^3, T = 173 K, Z = 4, 130185 reflections measured, radiation type MoK\$a$, radiation wavelength 0.71073 Å, final R indices R1 = 0.1226, wR2 = 0.2660, R indices (all data) R1 = 0.1348, wR2 = 0.2734.

---

1 G. M. Sheldrick, *Acta Cryst.* **2008**, *A64*, 112.

2 O. V. Dolomanov, L. J. Bourhis, R. J. Gildea, J. A. K. Howard, H. Puschmann, *J. Appl. Cryst.* **2009**, *42*, 339.

Computational Methods. DFT calculations are carried out in the *Gaussian* 09 package using the B3LYP or long-range corrected CAM-B3LYP exchange correlation functional.[3] The electronic structures were optimized using a general basis set with the Los Alamos effective core potential LANL2DZ basis set for Ru and 6-31G* for other atoms.[4] The solvation effects in CH_3CN are taken into account for all calculations with the conductor-like polarizable continuum model (CPCM).[5] No symmetry constraints were used in the optimization (nosymm keyword was used). Frequency calculations have been performed with the same level of theory to ensure the optimized geometries to be local minima. All orbitals have been computed at an isovalue of 0.02 e/bohr^3.

Synthesis. NMR spectra were recorded in the designated solvent on a Bruker Avance 400 MHz spectrometer. Spectra are reported in ppm values from residual protons of deuterated solvent. Mass data were obtained with a Bruker Daltonics Inc. Apex II FT-ICR or Autoflex III MALDI-TOF mass spectrometer. The matrix for MALDI-TOF measurement is α-cyano-4-hydroxycinnamic acid. Microanalysis was carried out using a Flash EA 1112 or Carlo Erba 1106 analyzer at the Institute of Chemistry, Chinese Academy of Sciences.

Synthesis of Ligand 1. A mixture of 3,5-bis(pyrid-2-yl)aniline[6] (50 mg, 0.20 mmol), 3,5-bis(pyrid-2-yl)bromobenze[7] (249 mg, 0.80 mmol), $Pd(dba)_2$ (8 mg, 0.008 mmol), tri-*tert*-butylphosphine ($P(t\text{-}Bu)_3$) (102 mg, 0.006 mmol) and NaO^tBu (58 mg, 0.6 mmol) in 12 mL dry toluene was bubbled with N_2 for 15 min, followed by heating at 120 ^oC for 3 days. After cooling down to room temperature, the pale yellow crude product precipitated out from the reaction mixture, which was collected by filtration and washing with water and ethyl ether to give 93 mg ligand **1** in 66% yield. This sample was pure enough for the next transformation without further purification. ^1H NMR (400 MHz, $CDCl_3$): δ 7.18-7.21 (m, 6H), 7.67-7.73 (m, 12H), 7.93 (s, 6H), 8.37 (s, 3H), 8.63 (d, *J* = 4.0 Hz, 6H). ^{13}C NMR (100 MHz, $CDCl_3$): δ 120.8, 121.0, 122.3, 123.7, 136.7, 141.5, 148.9, 149.6, 157.0. EI-HRMS calcd for $C_{48}H_{33}N_7$: 707.2808. Found: 707.2797.

Synthesis of Complex 2(PF$_6$)$_3$ and 2(PF$_6$)$_4$. To 20 mL dry acetone were added [Ru(tpy)Cl_3] (66 mg, 0.15 mmol) and AgOTf (116 mg, 0.45 mmol). The mixture was refluxed under N_2 atmosphere for 3 h. After cooling to room temperature, the mixture was filtered to remove the AgCl precipitate. The purple filtrate was concentrated to dryness under reduced pressure. To the residue were added ligand **1** (22 mg, 0.031 mmol), *N*-ethylmorpholine (0.5 mL), 6 mL dry DMF, and 6 mL dry *t*-BuOH. The mixture was bubbled with nitrogen for 10 minutes. The system was heated under microwave irradiation (powder = 600 W) for 30 min before cooling to room temperature. The solvent was removed under reduced pressure. The residue was subjected to column chromatography on silica gel (eluent: CH_3CN/H_2O/aq.KNO_3, 100/10/0.4), followed by anion exchange using KPF_6, to afford 17 mg of **2**$(PF_6)_3$ (25%) and 14 mg of **2**$(PF_6)_4$ (20%) as brown solids. Data of **2**$(PF_6)_3$: ^1H NMR (400 MHz, CD_3CN), δ 6.64 (t, *J* = 8.0 Hz, 6H), 7.07-7.11 (m, overlapped, 12H), 7.46 (d, *J* = 8.0 Hz, 6H), 7.56 (t, *J* = 8.0 Hz, 6H), 7.72 (t, *J* = 8.0 Hz, 6H), 8.13 (d, *J* = 8.0 Hz, 6H), 8.25 (t, *J* = 8.0 Hz, 3H), 8.46 (d, *J* = 8.0 Hz, 6H), 8.60 (s, 6H), 8.77 (d, *J* = 8.0

---

[3] T. Yanai, D. P. Tew, N. C. Handy, *Chem. Phys. Lett.* **2004**, *393*, 51.
[4] P. J. Hay, W. R. Wadt, *J. Chem. Phys*. **1985**, *82*, 299.
[5] V. Barone, M. Cossi, *J. Phys. Chem. A* **1998**, *102*, 1995.
[6] Z.-L. Gong, Y.-W. Zhong, *Organometallics* **2013**, *32*, 7495.
[7] C.-J. Yao, R.-H. Zheng, Q. Shi, Y.-W. Zhong, J. Yao, *Chem. Commun.* **2012**, *48,* 5680.

Hz, 6H). MALDI-MS, 1996.6 for $[M - PF_6 - 3H]^+$; 1850.7 for $[M - 2PF_6 - 3H]^+$. Anal. Calcd for $C_{93}H_{63}F_{18}N_{16}P_3Ru_3$: C, 52.09; H, 3.30; N, 9.99. Found: C, 52.13; H, 2.96; N, 10.46. Data of **2**$(PF_6)_4$: MALDI-MS, 1998.1 for $[M - 2PF_6]^+$; 1519.2 for $[M - Ru(tpy) - 3PF_6]^+$; 1374.2 for $[M - Ru(tpy) - 4PF_6]^+$; 1041.3 for $[M - 2Ru(tpy) - 4PF_6]^+$. Anal. Calcd for $C_{93}H_{63}F_{24}N_{16}P_4Ru_3$: C, 48.46; H, 3.07; N, 9.53. Found: C, 48.83; H, 2.78; N, 9.80.

Synthesis of Complex 3$(PF_6)_4$. Following the similar procedure for the synthesis of **2**$(PF_6)_3$, complex **3**$(PF_6)_4$ was prepared from $[Ru(vtpy)Cl_3]$[8] (70 mg, 0.15 mmol) and ligand **1** (22 mg, 0.031 mmol) in 34% yield (25 mg product was isolated). MALDI-MS (m/z): 2076.7 for $[M - 2PF_6]^+$; 1929.7 for for $[M - 3PF_6]^+$; 1786.7 for $[M - 4PF_6]^+$; 1067.6 for $[M - 2Ru(vtpy) - 4PF_6]^+$. Anal. Calcd for $C_{99}H_{69}F_{24}N_{16}P_4Ru_3 \cdot H_2O$: C, 49.62; H, 3.49; N, 9.48. Found: C, 49.84; H, 3.76; N, 9.39.

[8] C.-J. Yao, Y.-W. Zhong, H.-J. Nie, H. D. Abruña, J. Yao, *J. Am. Chem. Soc.* **2011**, *133*, 20720.

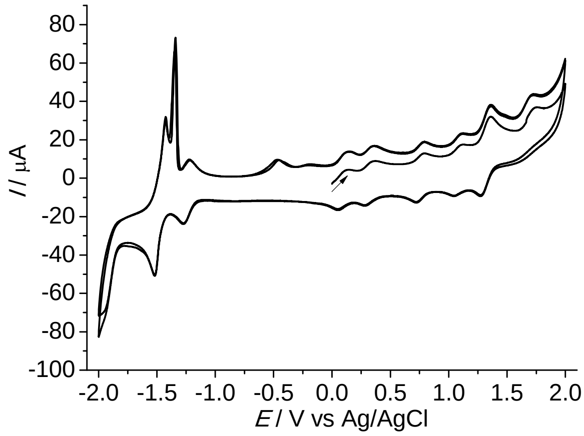

Figure S1. CV of **2**$(PF_6)_3$ in 0.1 M Bu_4NClO_4/CH_3CN. The high peaks around −1.4 V are caused by adsorption/desorption.

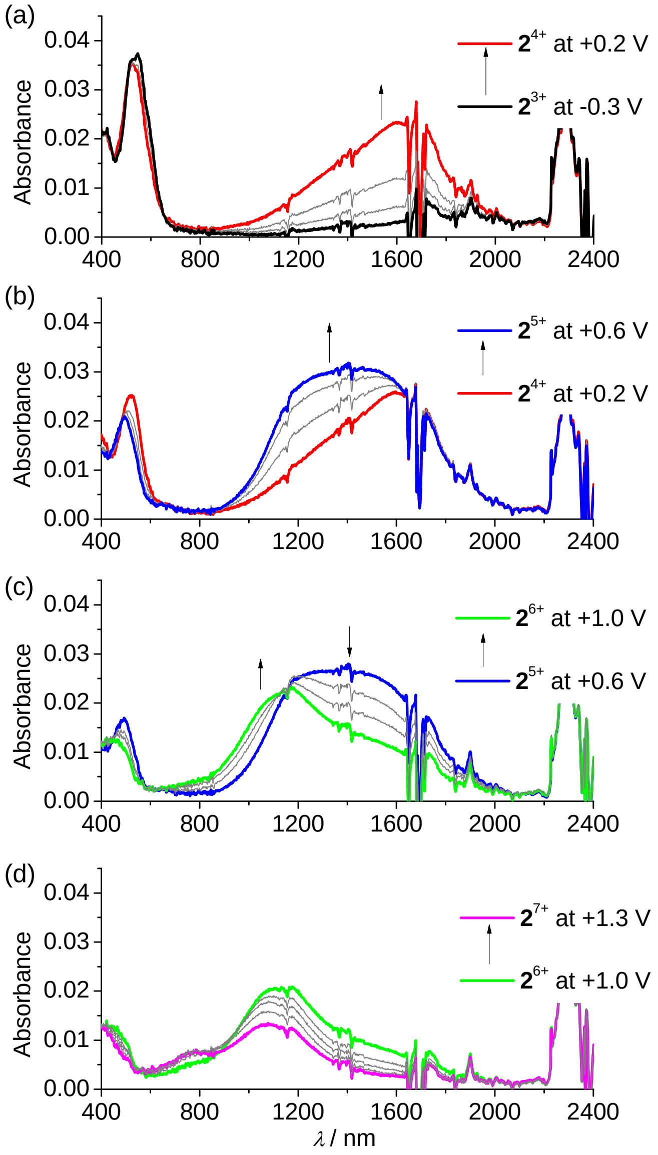

Figure S2. Absorption spectral changes of **2**$(PF_6)_3$ upon by stepwise electrolysis at a transparent ITO glass electrode in CH_3CN. The applied potentials were referenced versus Ag/AgCl. The quadruple-oxidation shown in (d) is very likely incomplete due to the high oxidation potential involved.

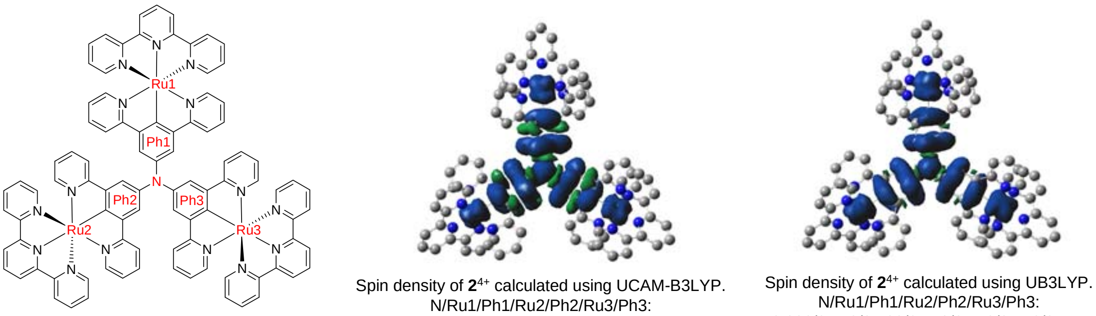

Spin density of **2**$^{4+}$ calculated using UCAM-B3LYP.
N/Ru1/Ph1/Ru2/Ph2/Ru3/Ph3:
0.331/0.070/0.154/0.071/0.156/0.074/0.160

Spin density of **2**$^{4+}$ calculated using UB3LYP.
N/Ru1/Ph1/Ru2/Ph2/Ru3/Ph3:
0.236/0.110/0.139/0.113/0.140/0.116/0.144

Figure S3. Comparison of the spin density distributions of **2**$^{4+}$ calculated using UCAM-B3LYP or UB3LYP.

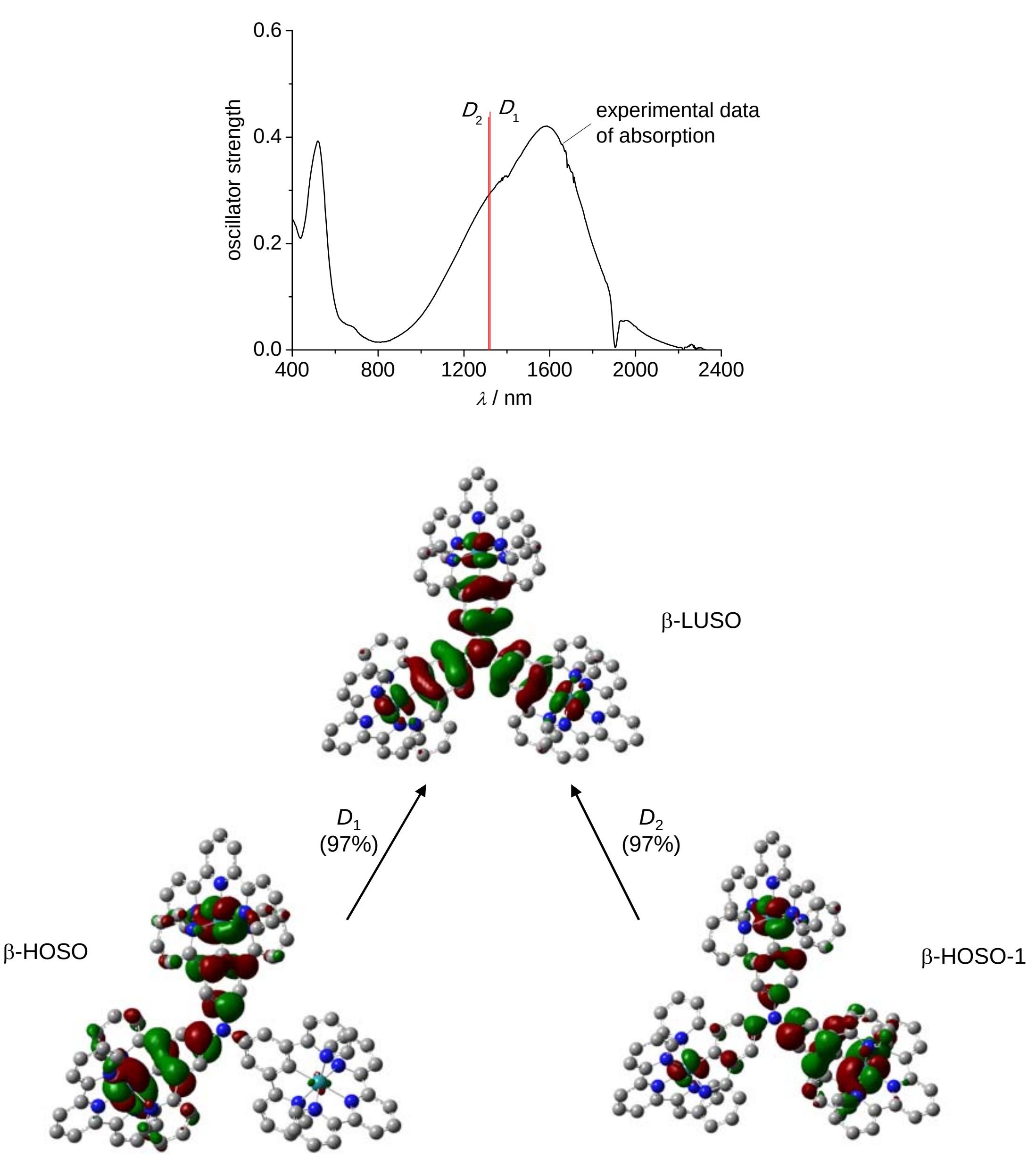

Figure S4. TDDFT-predicted NIR excitations (vertical bars) of $\mathbf{2}^{4+}$, along with the experimental data of absorption for comparison. D_1 and D_2 mean the first and second calculated excitations in the doublet state.

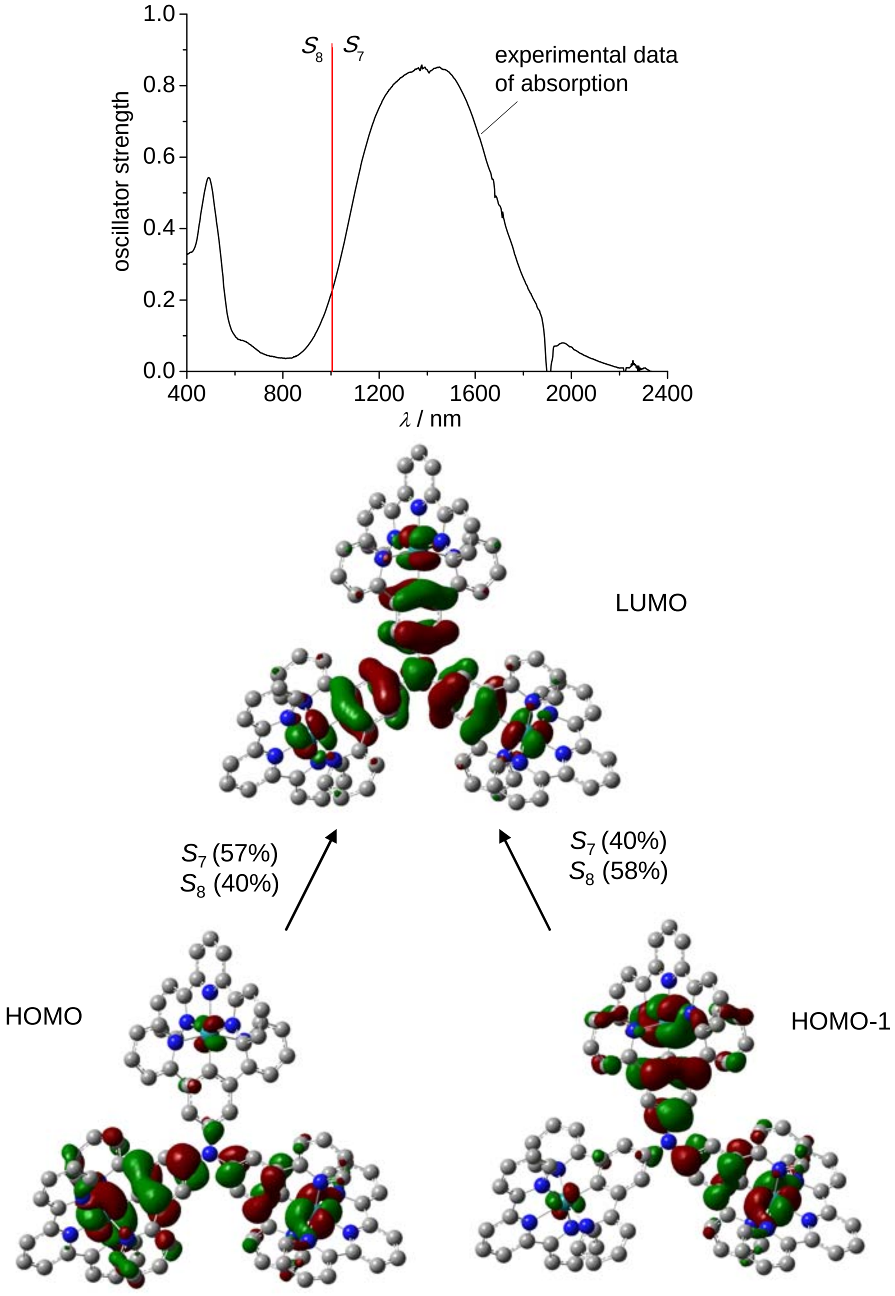

Figure S5. TDDFT-predicted NIR excitations (vertical bars) of singlet $\mathbf{2}^{5+}$, along with the experimental data of absorption for comparison. Other predicted excitations in the low-energy region have negligible oscillator strength. Depending on the spin directions, two possible spin states, singlet and triplet, are possible for complex $\mathbf{2}^{5+}$. The closed-shell singlet state was calculated to be 0.271 eV lower in energy relative to the triplet state, in accordance with the EPR-inactive nature of this complex. The TDDFT results of singlet $\mathbf{2}^{5+}$ show that the predicted S_7 and S_8 excitations are mainly responsible for the observed NIR absorptions. Both excitations are dominated by the HOMO → LUMO and HOMO-1 → LUMO transitions. The HOMO and HOMO-1 are dominated by ruthenium components. The LUMO is delocalized over the N(phenyl-Ru)$_3$ framework.

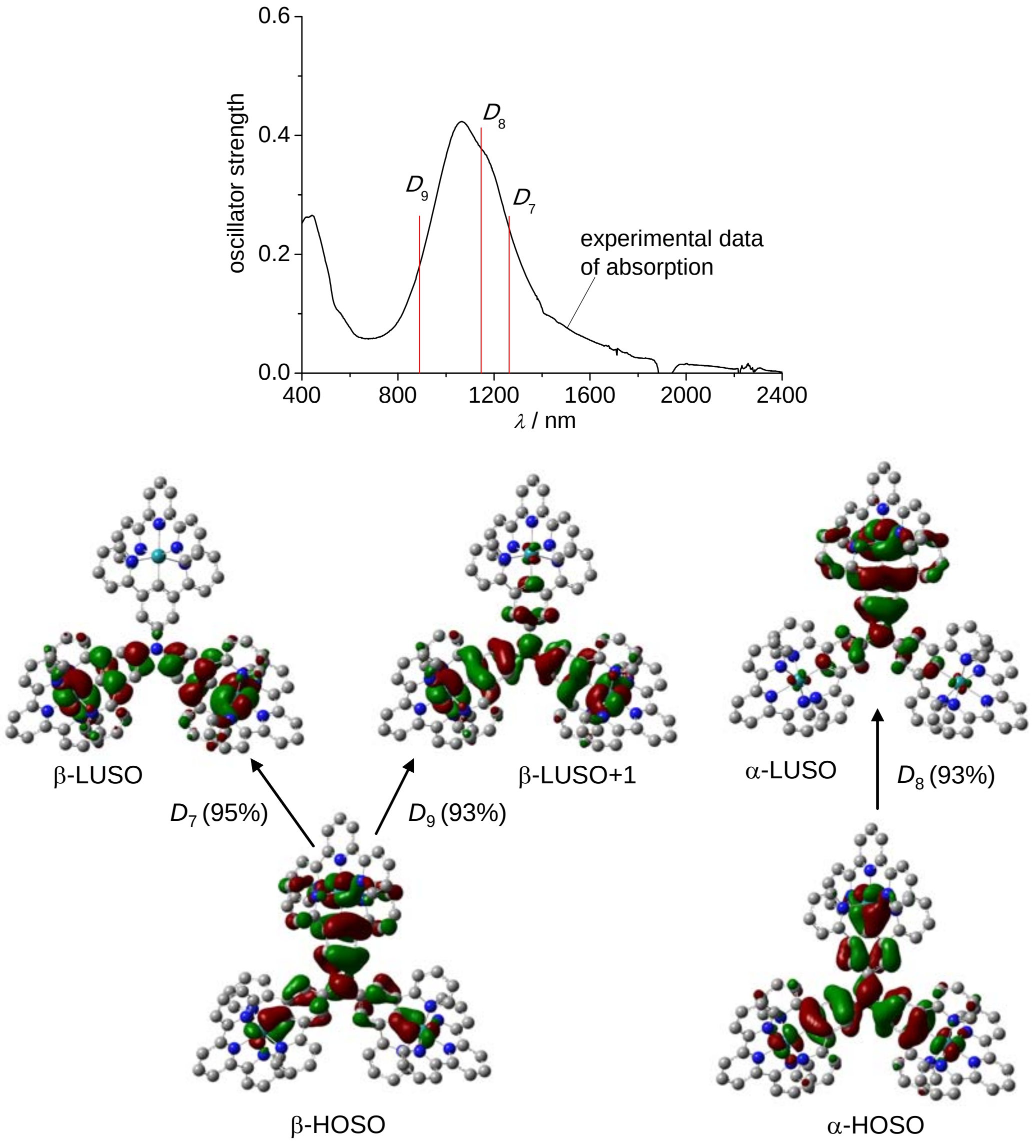

Figure S6. TDDFT-predicted NIR excitations (vertical bars) of doublet $\mathbf{2}^{6+}$, along with the experimental data of absorption for comparison. For $\mathbf{2}^{6+}$ with three free spins, doublet or quartet states are possible. DFT calculations show that the doublet state is 0.144 eV lower in energy relative to the quartet state. The TDDFT results of $\mathbf{2}^{6+}$ in the doublet state are given in Figure S6. The NIR absorptions could be predicted by the D_7, D_8, and D_9 excitations, which are dominated by the *β*–HOSO → *β*–LUSO, *α*–HOSO → *α*–LUSO, and *β*–HOSO → *β*–LUSO+1 transitions, respectively. The natures of these excitations are complex. Some ruthenium-to-ruthenium and amine-to-ruthenium charge transfer transition are involved.

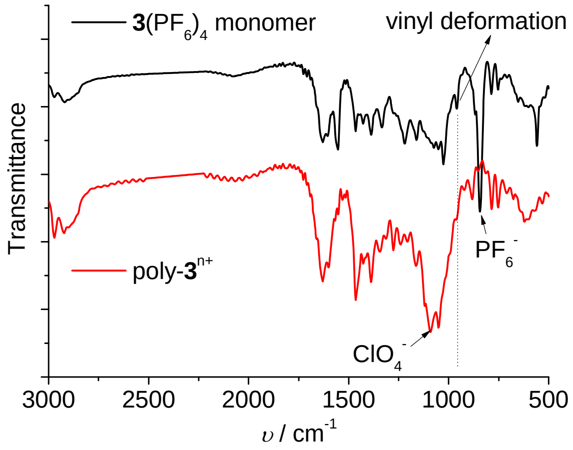

Figure S7. FTIR spectra of monomer **3**(PF_6)$_4$ (black curve) and poly-**3**$^{n+}$ (red curve). The polymer sample was obtained by scratching the polymer from the poly-**3**$^{n+}$/ITO film. The intense peak at 840 cm^{-1} of **3**(PF_6)$_4$ is attributed to the PF_6^- stretches. The signal around 1100 cm^{-1} of poly-**3**$^{n+}$ is assigned to the ClO_4^- anions. The weak peak at 960 cm^{-1} of **3**(PF_6)$_4$ is caused by the out-of-plane deformation vibration of vinyl groups. This peak is essentially absent in the polymer sample, which suggests that all of the three vinyl groups of the monomer are consumed during the polymerization. FTIR Measurements were performed in KBr pellets using a Bruker Tensor-27 spectrometer.

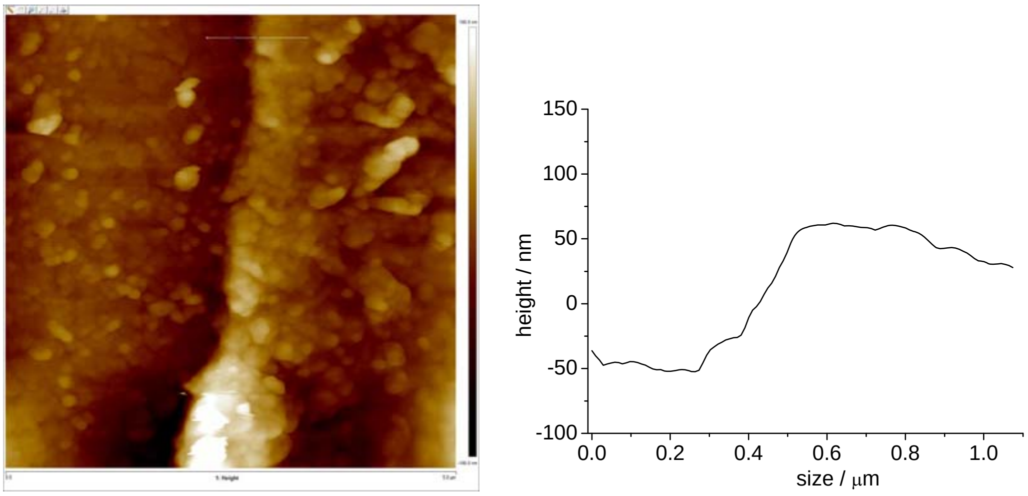

Figure S8. Estimating the thickness of the poly-3^{n+}/ITO film by measuring the step height produced by scanning across a scratching edge using AFM.

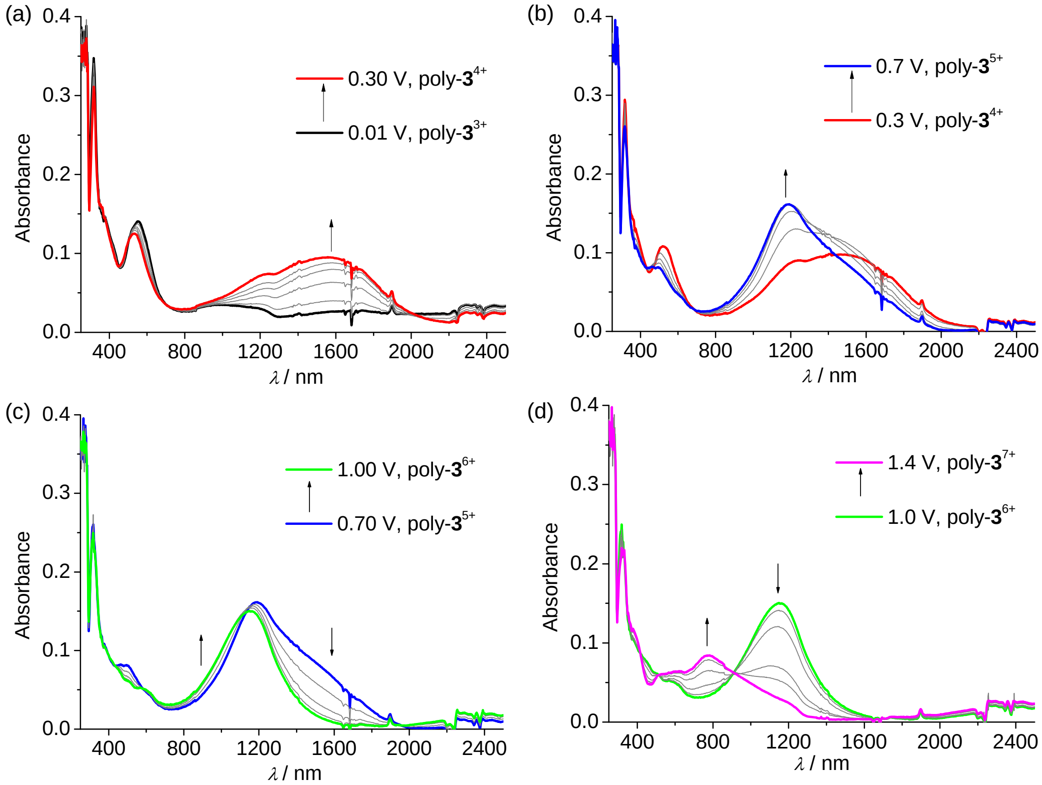

Figure S9. Absorption spectral changes of poly–3^{n+}/ITO film (Γ = 5.0 × 10^{-9} mol/cm^2) during spectroelectrochemical measurements. The applied potential was referenced versus Ag/AgCl.

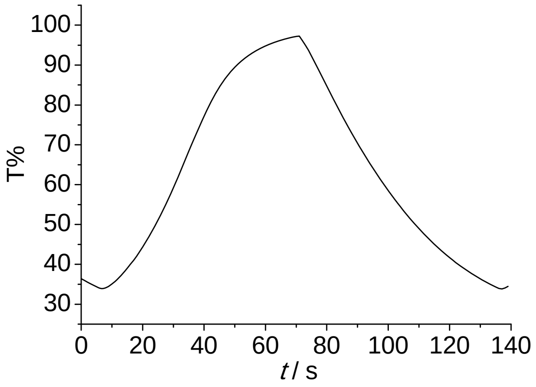

Figure S10. An enlarged plot of the transmittance changes monitored at λ = 1550 nm as a function of time during the electrochromic switching of the poly–**3**$^{n+}$/ITO film (Γ = 2.0 × 10^{-8} mol/cm^2) between −0.1 and +0.3 V in 0.1 M Bu_4NClO_4/CH_3CN. The interval is 65 s.

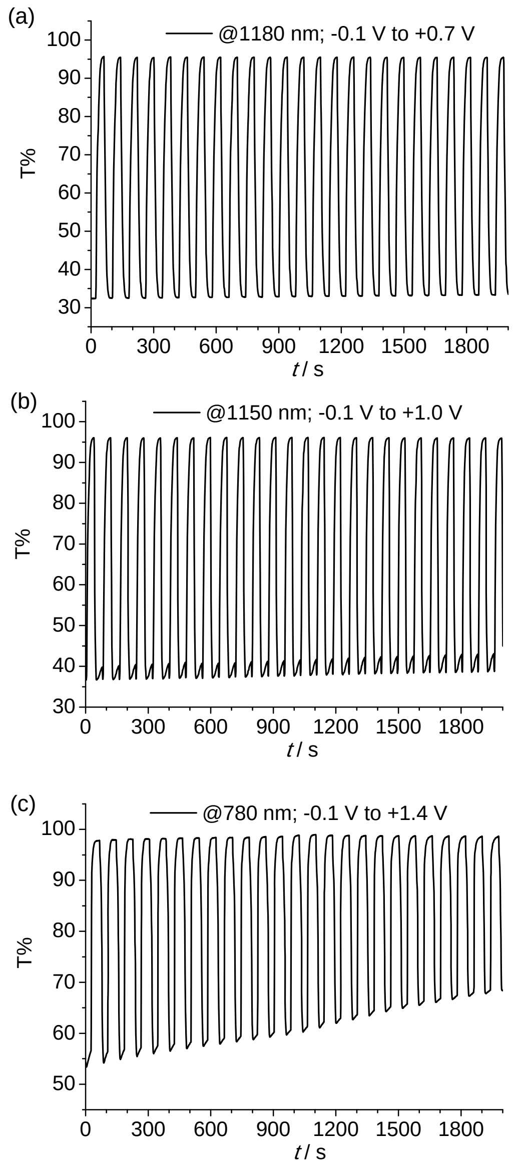

Figure S11. Transmittance changes monitored at λ = 1180 nm (a), 1150 nm (b), and 780 nm (c) as a function of time during the electrochromic switching of the poly–**3**$^{n+}$/ITO film (Γ = 2.0 × 10^{-8} mol/cm^2) between (a) −0.1 and +0.7 V, (b) −0.1 and +1.0 V, and (c) −0.1 and +1.4 V in 0.1 M Bu_4NClO_4/CH_3CN. The interval is 65 s.

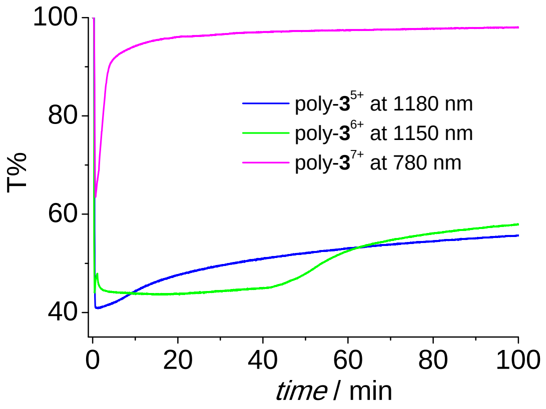

Figure S12. Optical memory test of the poly–**3**$^{n+}$/ITO film (Γ = 1.0 × 10^{-9} mol/cm^2) at different redox states.

^{1}H NMR spectrum of ligand **1** in $CDCl_3$:

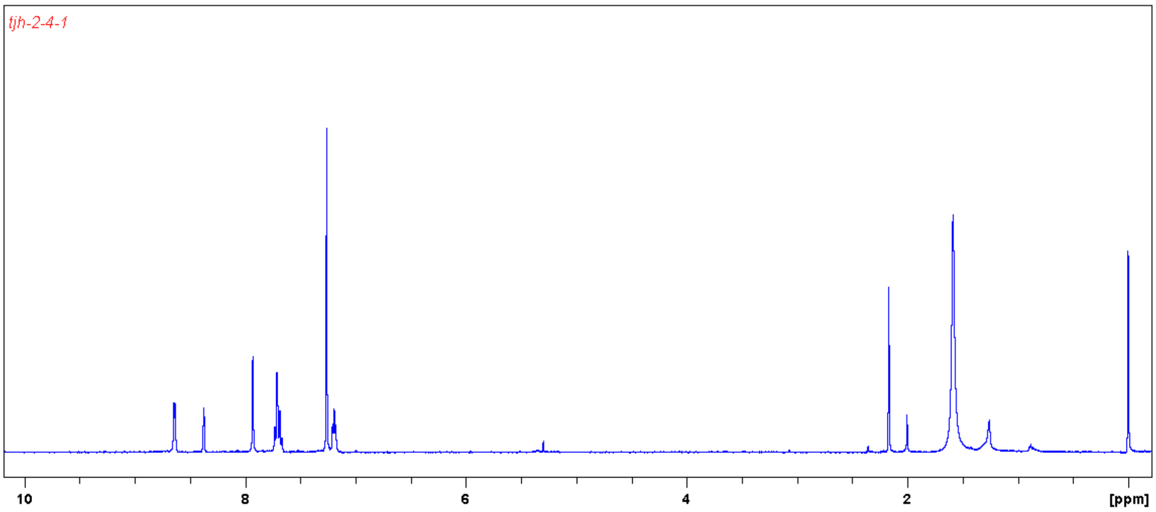

^{13}C NMR spectrum of ligand **1** in $CDCl_3$:

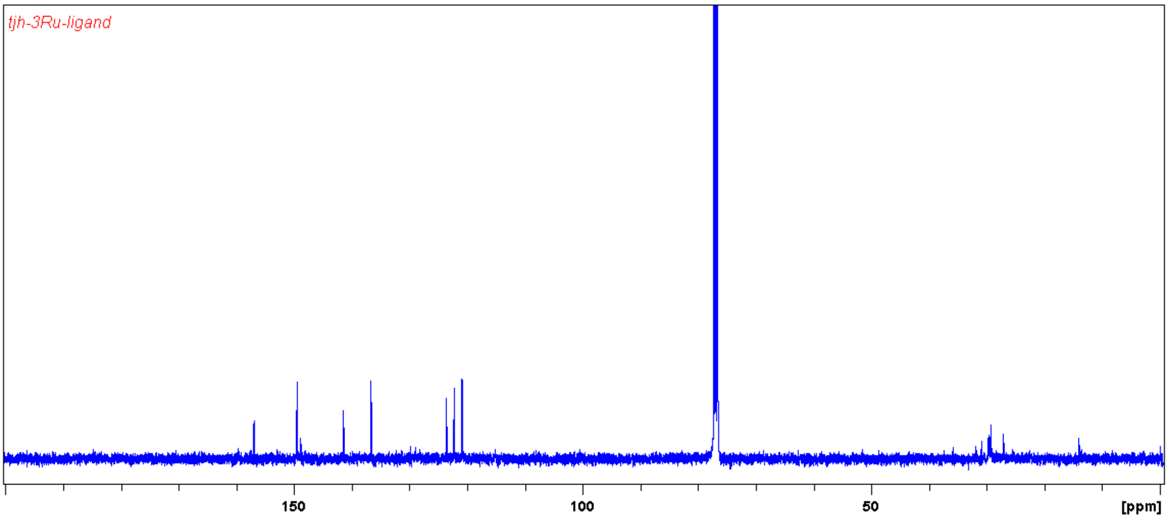

EI-Mass spectrum of ligand **1**:

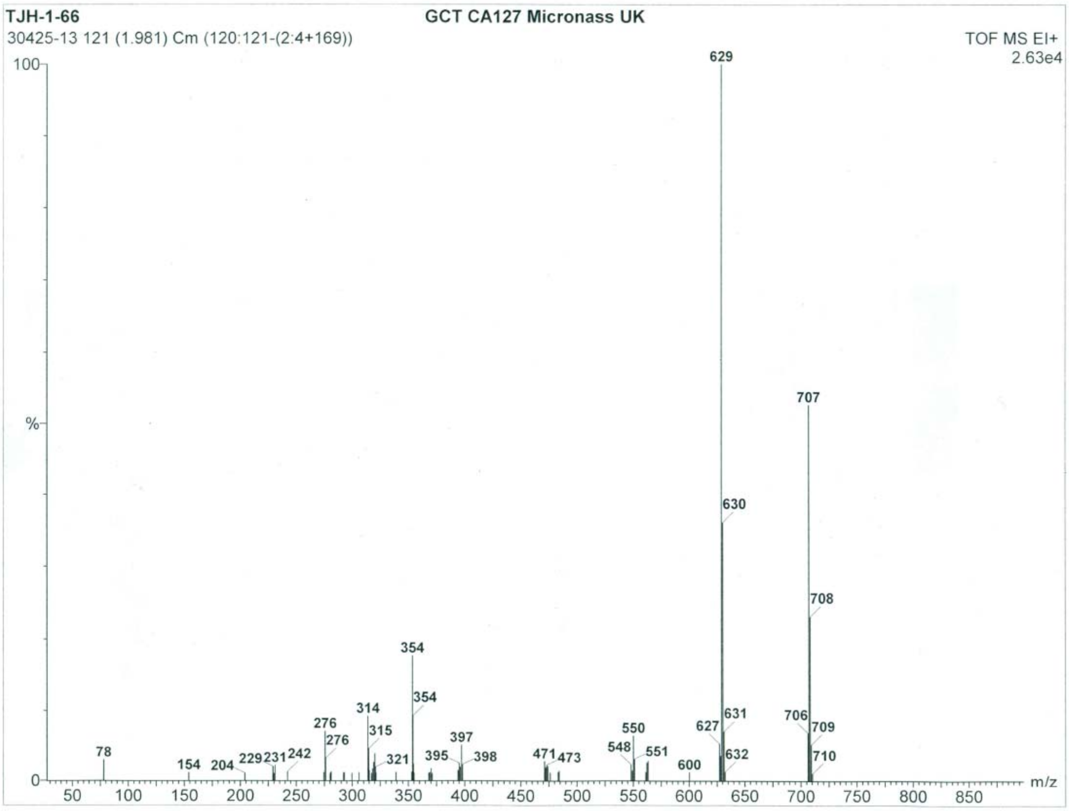

MALDI-TOF mass spectrum of **2**$(PF_6)_3$:

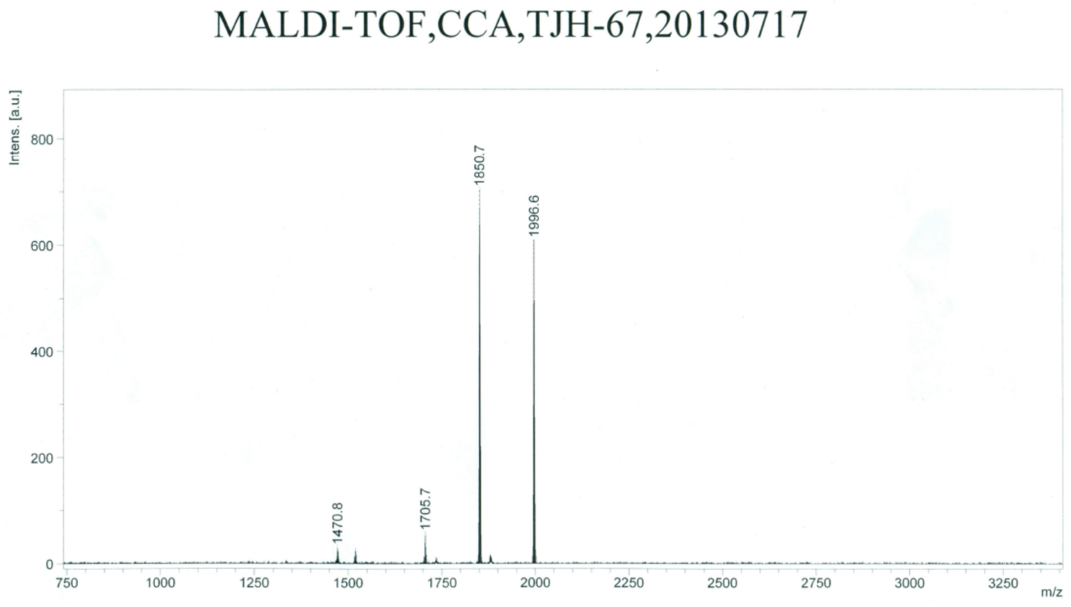

^1H NMR spectrum of **2**$(PF_6)_3$ in CD_3CN in the presence of small amount of aqueous hydrazine:

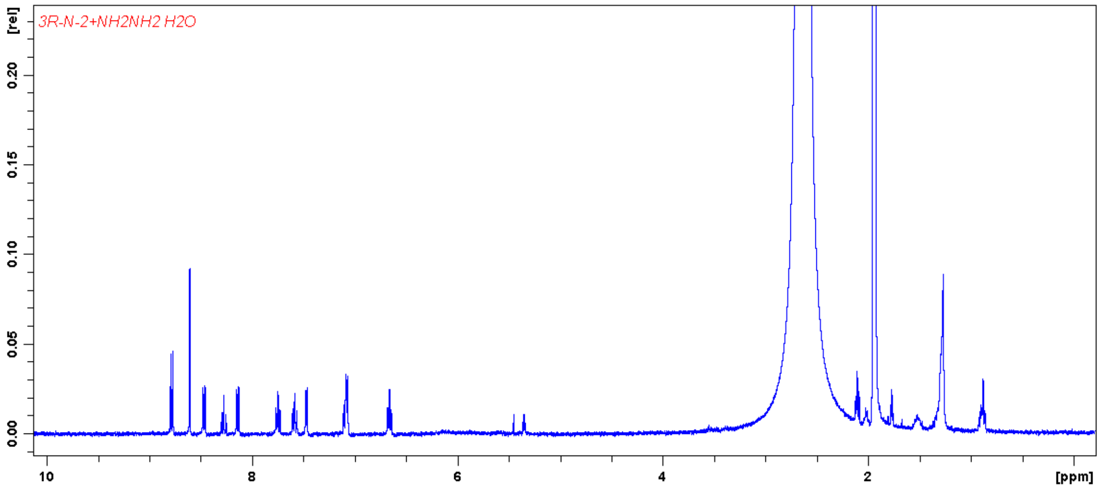

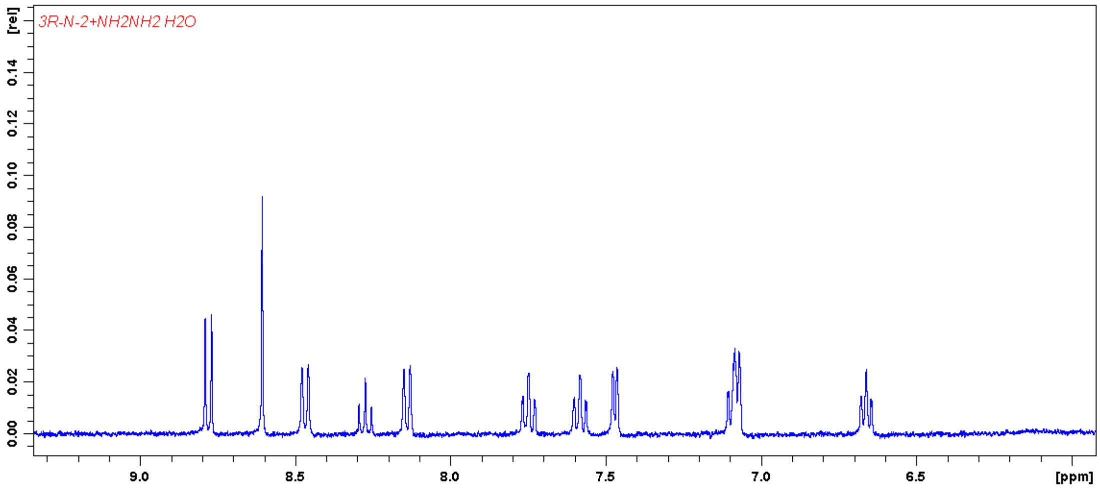

MALDI-TOF mass spectrum of **2**$(PF_6)_4$:

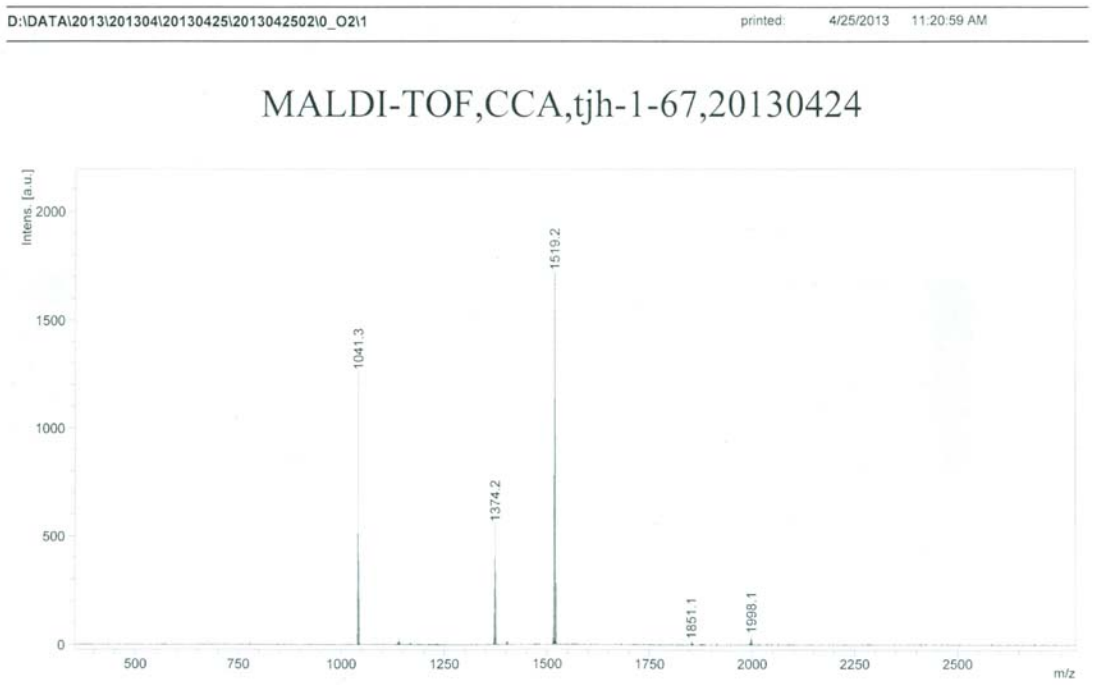

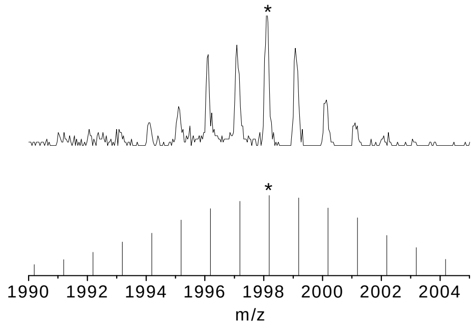

A comparison of the experimental (up) and theoretical (down) isotope distribution for $C_{93}H_{63}N_{16}F_{12}P_2Ru_3$, [**2**$(PF_6)_2]^+$ (after the loss of two anions from **2**$(PF_6)_4$). Calcd: 1998.18847. Found: 1998.1.

MALDI-TOF mass spectrum of complex **3**(PF_6)$_4$:

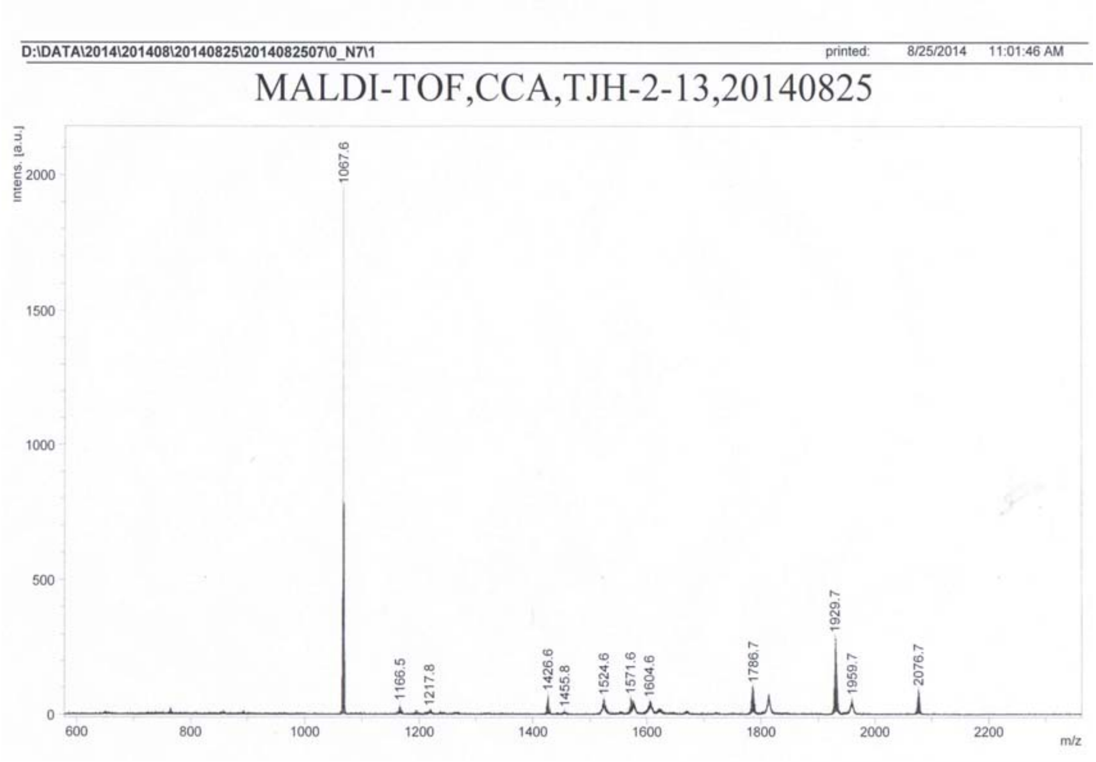

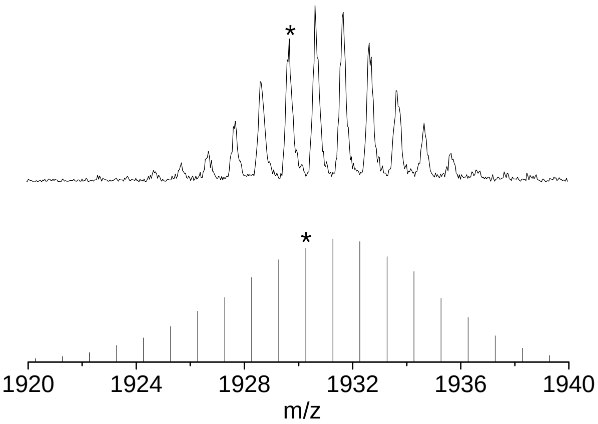

Experimental (up) and theoretical (bottom) isotope distribution for [M − 3PF$_6$], $C_{99}H_{69}N_{16}F_6PRu_3$. Calcd: 1930.27146. Found: 1929.7.

Cartesian coordinates of DFT-optimized structures of $\mathbf{2}^{4+}$ using UCAM-B3LYP:

Ru	4.11759697	14.09970290	22.75426028
N	5.43131018	14.61445266	21.16095574
N	5.49457914	12.53742243	22.76322507
N	3.43617807	12.87515447	24.35181729
N	2.62991832	13.34576238	21.40922189
N	5.03353399	15.49563967	24.09582039
N	0.01051475	18.66061969	22.77654975
C	5.35079004	15.69391075	20.37418982
H	4.52775273	16.36675486	20.58119973
C	6.25482481	15.95076439	19.35411071
H	6.14110145	16.84125222	18.74764501
C	7.28839159	15.05103289	19.13813125
C	7.38073302	13.92829974	19.94895780
H	8.17922550	13.21281771	19.79822060
C	6.44404590	13.72606301	20.95507986
C	6.48164817	12.54932045	21.85810178
C	7.41282286	11.51517423	21.83582924
H	8.22123583	11.49854695	21.11606246
C	7.28468788	10.48975096	22.76692772
C	6.24949388	10.50166221	23.69602803
H	6.15707942	9.69927657	24.41694895
C	5.34919404	11.56252395	23.67019413
C	4.18603705	11.75864603	24.57024790
C	3.86293835	10.86673577	25.58574539
H	4.46932997	9.98449199	25.74741063
C	2.76005941	11.11231370	26.39209827
C	2.00172830	12.25104493	26.16264226
H	1.13246154	12.48548898	26.76523438
C	2.37481732	13.10335475	25.13431010
H	1.81413867	14.00442401	24.91732414
C	2.65184372	12.17135243	20.76739233
H	3.51634736	11.54447160	20.95403897
C	1.64231323	11.76072200	19.91179686
H	1.71165345	10.79900479	19.41781677
C	0.55817828	12.60706798	19.71256691
H	-0.25259641	12.32191553	19.05086463
C	0.52461680	13.82570762	20.37281555
H	-0.31117474	14.50123519	20.23346783
C	1.56922169	14.18504596	21.22034636
C	1.64744625	15.44660928	21.97782073
C	0.71943764	16.47375608	21.97909234
H	-0.18017639	16.43688936	21.37437821
C	0.95776796	17.61819396	22.76601331
C	2.12627335	17.73305347	23.54524056
H	2.25814273	18.61933117	24.15655406
C	3.05213668	16.70365978	23.53346902
C	2.82131644	15.55199546	22.75249177
C	4.31645966	16.64195190	24.28805037
C	4.79007436	17.63898692	25.13666970
H	4.21055383	18.54351489	25.27900092
C	5.99916235	17.47042386	25.79396447
H	6.37318800	18.24343593	26.45658910
C	6.72097917	16.30043413	25.59055032
H	7.67033850	16.12498753	26.08213482
C	6.20003909	15.34356207	24.73451828
H	6.72789816	14.41562436	24.54531201
N	3.27464936	23.60791526	21.41995519

N	0.19478644	24.59388307	24.09589976
C	4.27355344	24.22346852	20.77598035
H	4.36838465	25.28841974	20.95541747
C	5.14478663	23.56021099	19.92692127
H	5.93645643	24.10835765	19.43045776
C	4.97315646	22.19415738	19.73751445
H	5.63479630	21.63887476	19.08147965
C	3.94186673	21.54633965	20.40018459
H	3.78999074	20.48148210	20.26910527
C	3.09622455	22.26605934	21.23996031
C	1.96849544	21.69411240	21.99687905
C	1.55116139	20.37435598	21.99512641
H	2.03836589	19.61746953	21.38994555
C	0.43879695	20.00130878	22.77640529
C	-0.25281178	20.95144494	23.55475676
H	-1.08713751	20.61733936	24.16192732
C	0.16665855	22.27057216	23.54486696
C	1.28005066	22.65383302	22.76823373
C	-0.42691322	23.39381596	24.29188638
C	-1.53006310	23.29791476	25.13586165
H	-2.01476829	22.33952819	25.28010531
C	-2.00320947	24.42737798	25.78601146
H	-2.86199165	24.35882677	26.44501176
C	-1.36237741	25.64324738	25.58030068
H	-1.69653856	26.55186535	26.06657383
C	-0.27033814	25.67811222	24.72815968
H	0.26040812	26.60370250	24.53609004
N	-5.91508296	19.00588192	21.45352249
N	-5.20123714	15.81580302	24.08670795
C	-6.95351182	19.56738714	20.82268818
H	-7.92072718	19.11164911	21.00196285
C	-6.82389091	20.66489440	19.98691516
H	-7.69897757	21.07929945	19.50092194
C	-5.55830743	21.20667401	19.79686009
H	-5.41545285	22.06613406	19.15078071
C	-4.47560601	20.63353906	20.44613623
H	-3.47982726	21.04008689	20.31438591
C	-4.66699090	19.53024048	21.27349375
C	-3.60100832	18.83614779	22.01780377
C	-2.25061571	19.14027971	22.01300466
H	-1.84502252	19.94859637	21.41425013
C	-1.36424858	18.35867274	22.78146422
C	-1.83343936	17.27451556	23.55055985
H	-1.12185259	16.71470160	24.14770304
C	-3.18419603	16.97266651	23.54380226
C	-4.07982694	17.74948548	22.77934011
C	-3.85138589	15.88529828	24.28172935
C	-3.20957114	14.97513564	25.11732668
H	-2.13773532	15.04169767	25.26224293
C	-3.94344950	13.98918846	25.75892552
H	-3.44904819	13.27777580	26.41156618
C	-5.31640940	13.92730912	25.55280622
H	-5.93032803	13.17419623	26.03198998
C	-5.90003475	14.85939399	24.70993005
H	-6.96707993	14.85003496	24.51848748
Ru	1.86372682	24.51046833	22.75489847
N	0.74159588	25.34761880	21.15410765
N	2.49080926	26.49693834	22.73503950

N	3.27102608	24.58157494	24.34754260
C	-0.14313927	24.70874629	20.37948053
H	-0.29077919	23.65871469	20.60032155
C	-0.83596405	25.33559642	19.35457216
H	-1.54127689	24.76908466	18.75854606
C	-0.60347956	26.68295737	19.12023712
C	0.31281361	27.35438276	19.91783847
H	0.51013269	28.40598972	19.75266744
C	0.97603145	26.67107417	20.92974973
C	1.96675710	27.32363862	21.82079320
C	2.36961762	28.65509462	21.77992008
H	1.96407470	29.34609797	21.05186585
C	3.31547433	29.08851618	22.70313964
C	3.84359322	28.20898920	23.64254409
H	4.57942530	28.55492136	24.35725882
C	3.40286213	26.88891528	23.63423336
C	3.84171411	25.80310177	24.54533159
C	4.78115455	26.00014356	25.55004286
H	5.22619526	26.97641420	25.69453471
C	5.14690704	24.93970887	26.36760266
C	4.56138829	23.69945869	26.15965135
H	4.81470597	22.84218308	26.77166971
C	3.63048825	23.56521965	25.14045596
H	3.14719914	22.61707108	24.93902994
Ru	-5.97625056	17.31443511	22.76690497
N	-6.13504607	15.94477198	21.14742732
N	-8.00546327	16.84332077	22.75153184
N	-6.74431694	18.46822408	24.37929217
C	-5.13929112	15.52096821	20.36020261
H	-4.15893047	15.92467066	20.58179241
C	-5.33198732	14.62198338	19.32189334
H	-4.48883280	14.31331429	18.71568958
C	-6.61135851	14.13975950	19.08749269
C	-7.65092193	14.57295723	19.89888334
H	-8.65709584	14.20909389	19.73384884
C	-7.39467948	15.47552776	20.92386052
C	-8.45514910	15.98134342	21.83021150
C	-9.80535808	15.64557358	21.79754697
H	-10.19805417	14.95219739	21.06475026
C	-10.65350274	16.22326315	22.73658682
C	-10.16020424	17.11501413	23.68331349
H	-10.82729355	17.55913619	24.41118886
C	-8.80094262	17.41344523	23.66591308
C	-8.08482610	18.33344634	24.58373557
C	-8.72736211	19.02861590	25.60084599
H	-9.79332089	18.91171483	25.74952522
C	-7.99695516	19.87366117	26.42485593
C	-6.63252772	20.00413035	26.21114823
H	-6.02045591	20.65071563	26.82834935
C	-6.04841552	19.28469959	25.17932668
H	-4.98722410	19.35372177	24.97373761
H	-8.49032193	20.41988481	27.22088880
H	-11.70887691	15.97583575	22.73074629
H	-6.80239573	13.43586909	18.28531661
H	-1.12563939	27.20824094	18.32847313
H	3.64422358	30.12139397	22.69031133
H	5.87892640	25.08454945	27.15412251
H	8.01516415	15.21643200	18.35081340

H	7.99803204	9.67349346	22.76851631
H	2.50075991	10.42198822	27.18688916

Cartesian coordinates of DFT-optimized structures of $\mathbf{2}^{4+}$ using UB3LYP:

Ru	4.13700086	14.08267546	22.76570723
N	5.45614383	14.61185969	21.17763122
N	5.52425715	12.52133592	22.76484943
N	3.46148325	12.83795024	24.35409646
N	2.65069884	13.33320907	21.40664538
N	5.04422430	15.47689648	24.12478707
N	0.00433764	18.65938740	22.79377373
C	5.37249811	15.70298550	20.39441492
H	4.54670272	16.37135827	20.60580070
C	6.27934096	15.97209314	19.37365767
H	6.16128919	16.86809124	18.77447276
C	7.32197101	15.07487661	19.14861240
C	7.41887246	13.94246635	19.95284498
H	8.22120550	13.23137981	19.79567896
C	6.48016018	13.72280942	20.96370444
C	6.52102563	12.54225382	21.85628062
C	7.46030291	11.50636660	21.83027150
H	8.27091001	11.49915852	21.11157350
C	7.33568135	10.46906849	22.75669301
C	6.29427159	10.46999360	23.68699503
H	6.20469331	9.66132741	24.40254104
C	5.38289385	11.53050761	23.66943153
C	4.21982606	11.71386922	24.56702278
C	3.89130287	10.81126535	25.58156912
H	4.49953947	9.92833030	25.73806885
C	2.78286818	11.04858759	26.39026975
C	2.01947565	12.19307198	26.16632427
H	1.14779798	12.42175704	26.76935725
C	2.39269757	13.05752821	25.14193232
H	1.82954737	13.95888624	24.93151980
C	2.67530624	12.15610350	20.75653673
H	3.53908367	11.52795455	20.94349447
C	1.66512059	11.74814292	19.89165812
H	1.73767632	10.78761753	19.39371974
C	0.57651777	12.59803720	19.68975858
H	-0.23161001	12.31510248	19.02252724
C	0.53901772	13.81796592	20.35782237
H	-0.29779295	14.49324630	20.21704661
C	1.58271531	14.17909997	21.21682273
C	1.65783950	15.43328719	21.98270572
C	0.72730836	16.46884855	21.98306598
H	-0.16636467	16.43544291	21.36832294
C	0.95594077	17.61117972	22.78440089
C	2.12121742	17.71805997	23.57818164
H	2.24807675	18.59945321	24.19865863
C	3.05696312	16.68695345	23.56503661
C	2.83164208	15.53505851	22.77043707
C	4.31553677	16.62622015	24.32515289
C	4.78692737	17.62042328	25.18936972
H	4.20293115	18.52195689	25.33864708
C	5.99882346	17.45085150	25.85173115
H	6.36729027	18.21997539	26.52329423
C	6.72949728	16.28089368	25.63861454

H	7.67896144	16.10484001	26.13191193
C	6.21497745	15.32472511	24.76916716
H	6.74917246	14.40129546	24.57413788
N	3.27328153	23.62305190	21.39390173
N	0.21934488	24.61874070	24.11290471
C	4.27063512	24.23855775	20.73434807
H	4.37209803	25.30322110	20.91340207
C	5.13111142	23.56996505	19.86975690
H	5.91845726	24.11720878	19.36341227
C	4.95232004	22.19877392	19.67935723
H	5.60393203	21.64202945	19.01322743
C	3.92418892	21.55094344	20.35715502
H	3.76787448	20.48590893	20.22580900
C	3.08596164	22.27245455	21.21397480
C	1.96729822	21.70684779	21.98490006
C	1.54046821	20.38173705	21.98778371
H	2.01745917	19.62478376	21.37348676
C	0.43713277	20.00664087	22.78892716
C	-0.24074522	20.96167759	23.58158181
H	-1.06692115	20.62952298	24.20203928
C	0.17908060	22.28916936	23.56533734
C	1.28781015	22.67195565	22.76970384
C	-0.40496247	23.41048579	24.31878102
C	-1.50325184	23.32041268	25.18080779
H	-1.98842602	22.36250909	25.33284010
C	-1.96918081	24.45547184	25.83702484
H	-2.82088851	24.38895807	26.50669880
C	-1.32634767	25.67533917	25.62035935
H	-1.65387011	26.58617382	26.10915350
C	-0.24008380	25.70924451	24.75207908
H	0.28844430	26.63481262	24.55236584
N	-5.94242481	19.00200575	21.44626477
N	-5.23619995	15.80825717	24.09490083
C	-6.98333329	19.56676874	20.80894894
H	-7.95209543	19.11298369	20.98649621
C	-6.84878431	20.66704191	19.96854704
H	-7.72342201	21.08167133	19.47978825
C	-5.57695347	21.21000115	19.77912260
H	-5.43176744	22.06888991	19.13141139
C	-4.49240161	20.63486629	20.43419804
H	-3.49557135	21.04143714	20.30363382
C	-4.68398940	19.52778764	21.26787288
C	-3.62443657	18.83394662	22.01761613
C	-2.26508300	19.13446231	22.01529748
H	-1.85754541	19.93843045	21.41095775
C	-1.37774569	18.35760344	22.79616773
C	-1.85477263	17.27719235	23.57439765
H	-1.14683748	16.72079216	24.18010679
C	-3.21214996	16.96858285	23.56178688
C	-4.10854712	17.74543437	22.78583173
C	-3.87811827	15.88310437	24.29954304
C	-3.24012989	14.97089709	25.14713228
H	-2.16875110	15.03993054	25.30027914
C	-3.97858257	13.98131452	25.78867545
H	-3.48668363	13.27268231	26.44758203
C	-5.35572779	13.91515567	25.57125043
H	-5.97195073	13.16105666	26.04809861
C	-5.93937596	14.84655441	24.71868995

H	-7.00532309	14.83279865	24.52013577
Ru	1.87763332	24.53346018	22.74908270
N	0.74194418	25.37948490	21.15856237
N	2.51173355	26.52402671	22.72188797
N	3.30486849	24.60647749	24.32739412
C	-0.15690117	24.73914000	20.38817486
H	-0.30442194	23.68922239	20.61063135
C	-0.86093518	25.36910541	19.36656733
H	-1.57359421	24.80205590	18.77811181
C	-0.62837642	26.72222550	19.12653354
C	0.30028964	27.39483365	19.91630434
H	0.49762582	28.44663942	19.74689941
C	0.97866892	26.71192549	20.92869407
C	1.97810889	27.36022085	21.80763489
C	2.38791020	28.69659852	21.76461511
H	1.97637910	29.38932763	21.04032126
C	3.34816292	29.13021397	22.68095759
C	3.88520859	28.24522604	23.61808149
H	4.63010286	28.58946473	24.32549570
C	3.44065042	26.91926592	23.61681216
C	3.88570424	25.83500756	24.52144643
C	4.84100634	26.02424208	25.52299635
H	5.29138656	26.99947951	25.66438399
C	5.21320269	24.95853435	26.33814161
C	4.61815549	23.71494046	26.13314359
H	4.87499112	22.85496948	26.74158667
C	3.67284532	23.58333566	25.12056779
H	3.18516709	22.63625963	24.92360564
Ru	-6.01049595	17.30758777	22.76514381
N	-6.16647534	15.93570665	21.14294477
N	-8.04565169	16.83436731	22.73887175
N	-6.79332230	18.45915636	24.37560068
C	-5.16089479	15.50911041	20.35675782
H	-4.18132411	15.91157019	20.58502250
C	-5.34842472	14.60867431	19.31257035
H	-4.50002507	14.30068093	18.71166657
C	-6.63202929	14.12469165	19.06627888
C	-7.68015124	14.55832966	19.87383225
H	-8.68591711	14.19463941	19.70048458
C	-7.43400143	15.46372245	20.90884591
C	-8.49656770	15.96796254	21.80832240
C	-9.85377717	15.63281808	21.77016565
H	-10.24298873	14.93973844	21.03397526
C	-10.71090408	16.21121135	22.70855757
C	-10.21966715	17.10496294	23.66229875
H	-10.89116037	17.54875067	24.38760073
C	-8.85398058	17.40625117	23.65501187
C	-8.14451433	18.32305758	24.57598831
C	-8.78982487	19.02251576	25.59852560
H	-9.85714617	18.90585895	25.74375438
C	-8.06024212	19.86877348	26.42930234
C	-6.68850425	19.99879423	26.21931153
H	-6.07736146	20.64431512	26.84019911
C	-6.09775828	19.27879196	25.18541841
H	-5.03530329	19.34794575	24.98531379
H	-8.55658286	20.41446529	27.22491542
H	-11.76704572	15.96386549	22.69672151
H	-6.81762138	13.42186013	18.26083983

H	-1.15831469	27.24764607	18.33896073
H	3.67969174	30.16296926	22.66456299
H	5.95442158	25.09993268	27.11772104
H	8.04857497	15.25008487	18.36217600
H	8.05357025	9.65576857	22.75374444
H	2.52323164	10.35118415	27.17982433

Cartesian coordinates of DFT-optimized structures of **2**$^{5+}$ (singlet):

Ru	4.10570563	14.11074895	22.75642303
N	5.42561988	14.60516983	21.15422074
N	5.49703849	12.53079766	22.76194399
N	3.44759955	12.87446235	24.36248577
N	2.61845456	13.33413212	21.41369677
N	5.04573330	15.50137797	24.09619896
N	0.01158126	18.66089270	22.78295111
C	5.34030471	15.68986085	20.36245442
H	4.52050735	16.36569735	20.57129972
C	6.24163179	15.94455259	19.33296151
H	6.12301856	16.83545011	18.72661360
C	7.27793822	15.04072885	19.10937666
C	7.37551369	13.91527759	19.92360979
H	8.17347261	13.19923256	19.76784408
C	6.44311867	13.70973340	20.94219376
C	6.48437672	12.53759209	21.84582381
C	7.41760270	11.49625122	21.82092438
H	8.22166562	11.47710511	21.09544539
C	7.29401648	10.46918454	22.75833807
C	6.26095972	10.48500958	23.69729282
H	6.17202355	9.68371780	24.42086926
C	5.35672159	11.55146129	23.67616780
C	4.20205616	11.75014263	24.58127934
C	3.87800161	10.86070479	25.60775514
H	4.48373434	9.97717707	25.76927612
C	2.77681565	11.11220824	26.42241632
C	2.01698931	12.25688917	26.19179161
H	1.15110429	12.49682750	26.79849486
C	2.38565048	13.10877172	25.15516762
H	1.82326479	14.00931673	24.94162566
C	2.64273737	12.15279074	20.77444706
H	3.50866050	11.52752020	20.95943234
C	1.62542187	11.73713731	19.91984406
H	1.69639913	10.77309484	19.42883763
C	0.53424227	12.58194725	19.71925365
H	-0.27789759	12.29224562	19.06024747
C	0.49829380	13.80801511	20.37849145
H	-0.34130636	14.47971318	20.23844432
C	1.54807153	14.17413808	21.22466598
C	1.62862051	15.43450885	21.98300238
C	0.70178168	16.46166994	21.98981051
H	-0.19708174	16.42966355	21.38363125
C	0.95427329	17.62121472	22.77226633
C	2.13957706	17.74645451	23.54665617
H	2.26918073	18.63287128	24.15824515
C	3.06339532	16.71627516	23.54036520
C	2.82016529	15.54841299	22.75843755
C	4.32832215	16.65616533	24.29310491
C	4.80976535	17.65583482	25.14209631

H	4.23370521	18.56270238	25.28858421
C	6.02730532	17.48348792	25.79571911
H	6.40642028	18.25582392	26.45718628
C	6.74757440	16.30792088	25.58634373
H	7.70006545	16.13016870	26.07282960
C	6.22028446	15.34560988	24.72960894
H	6.74719688	14.41750818	24.53854277
N	3.29504930	23.60199916	21.42734722
N	0.17836718	24.60391273	24.08563695
C	4.30030250	24.22249616	20.78797060
H	4.39098414	25.28841567	20.96352690
C	5.18353269	23.55375238	19.94484075
H	5.97753736	24.10397136	19.45264214
C	5.01898357	22.18164483	19.75749840
H	5.68830125	21.62634607	19.10834381
C	3.98069185	21.52878758	20.41715478
H	3.83588653	20.46200514	20.28842839
C	3.12243706	22.25102034	21.24988704
C	1.99135803	21.68300264	22.00389186
C	1.57357416	20.36417011	22.00808765
H	2.05765219	19.60500312	21.40314868
C	0.44009553	19.99630710	22.78349880
C	-0.27032005	20.95624913	23.55490958
H	-1.10334009	20.62006568	24.16271638
C	0.14819134	22.27490496	23.54714698
C	1.28323809	22.65481475	22.77137953
C	-0.45073407	23.39951473	24.28696058
C	-1.56409672	23.31116139	25.12630068
H	-2.05375565	22.35513908	25.27443126
C	-2.04118190	24.45137929	25.76781353
H	-2.90516859	24.38916000	26.42157588
C	-1.39351066	25.66817120	25.55659836
H	-1.72931646	26.58175651	26.03419115
C	-0.29004545	25.69828985	24.70830268
H	0.24215715	26.62253147	24.51398170
N	-5.92063461	19.03043098	21.47081532
N	-5.19868592	15.79472155	24.07789821
C	-6.96761963	19.59819033	20.84939639
H	-7.93395169	19.13979149	21.02639099
C	-6.84014412	20.71096854	20.02261313
H	-7.71904789	21.12900275	19.54485069
C	-5.57277217	21.26085255	19.83260326
H	-5.43457552	22.12864064	19.19573241
C	-4.48107886	20.68092122	20.47378966
H	-3.48700461	21.09361095	20.34302117
C	-4.66743912	19.56340857	21.29125145
C	-3.60203155	18.86198143	22.02858179
C	-2.25205802	19.16462811	22.02810992
H	-1.84366389	19.97248073	21.43057165
C	-1.35865437	18.36117221	22.78822129
C	-1.82622437	17.25498300	23.54914780
H	-1.11321498	16.69652007	24.14589384
C	-3.17623915	16.95290290	23.54575905
C	-4.08082393	17.75133771	22.78496219
C	-3.84115422	15.86009283	24.27688748
C	-3.19989295	14.93759300	25.10746171
H	-2.12758368	14.99941794	25.25577279
C	-3.94024768	13.94258536	25.74077289

H	-3.44804098	13.22392947	26.38807960
C	-5.31747167	13.88530933	25.52949708
H	-5.93428411	13.12789836	25.99996761
C	-5.90369105	14.82972794	24.69133350
H	-6.97049492	14.82181965	24.49870134
Ru	1.85925607	24.49492200	22.75189835
N	0.75606040	25.34126211	21.13508320
N	2.49197729	26.50379558	22.72304951
N	3.25921588	24.59977393	24.35654205
C	-0.12696727	24.69164883	20.35438711
H	-0.27670718	23.64285540	20.57852685
C	-0.81529040	25.31324800	19.31689362
H	-1.51545927	24.73936252	18.72042974
C	-0.58336810	26.66497567	19.07248660
C	0.32944896	27.34603441	19.87380272
H	0.52602233	28.39732206	19.70153320
C	0.99225926	26.67206056	20.90127301
C	1.97389895	27.32947031	21.79339219
C	2.38081703	28.66654294	21.74615296
H	1.98162417	29.35212906	21.00854851
C	3.32247045	29.10907842	22.67691266
C	3.84392461	28.23353778	23.63113714
H	4.57442338	28.58517617	24.34950791
C	3.40177228	26.90678506	23.63104041
C	3.83238541	25.83054628	24.55216088
C	4.76718903	26.02976415	25.56994813
H	5.21214058	27.00702705	25.71300156
C	5.12628713	24.97065886	26.39982172
C	4.53879914	23.72461583	26.19277689
H	4.78543909	22.86954747	26.81196624
C	3.61331134	23.58249840	25.16332753
H	3.13348089	22.63187582	24.96660510
Ru	-5.95941161	17.31945277	22.77004071
N	-6.14017872	15.96630278	21.13149707
N	-8.01053343	16.84154401	22.75008713
N	-6.74812215	18.44516534	24.39963872
C	-5.13795677	15.55148899	20.33487528
H	-4.15670630	15.95098024	20.55903927
C	-5.33130700	14.66286878	19.28154379
H	-4.48594125	14.36391120	18.67214899
C	-6.61501174	14.17973577	19.03811972
C	-7.65899950	14.60218654	19.85723154
H	-8.66498767	14.23853139	19.68635772
C	-7.40768280	15.49564200	20.90033557
C	-8.46511722	15.98789929	21.81242913
C	-9.82216983	15.65193313	21.77705506
H	-10.21553443	14.96878017	21.03413979
C	-10.67320777	16.21778548	22.72809241
C	-10.17722835	17.09930402	23.69024465
H	-10.84425267	17.53328154	24.42523677
C	-8.81168598	17.40086743	23.67730034
C	-8.09719393	18.30639966	24.60558318
C	-8.73640501	18.99251559	25.63989636
H	-9.80273495	18.87412732	25.78961257
C	-8.00164378	19.82819474	26.47714330
C	-6.63176708	19.96063658	26.26123332
H	-6.01639617	20.59769482	26.88639316
C	-6.04646931	19.25392005	25.21498269

H	-4.98522492	19.32729619	25.01231437
H	-8.49339290	20.36371433	27.28231771
H	-11.72922156	15.97004877	22.71947772
H	-6.80462680	13.48590635	18.22598446
H	-1.10146503	27.18365023	18.27280622
H	3.65205239	30.14235214	22.65839532
H	5.85190216	25.11980826	27.19238144
H	7.99986214	15.20502103	18.31645144
H	8.00665064	9.65135692	22.75710715
H	2.52049916	10.42494384	27.22174083

Cartesian coordinates of DFT-optimized structures of **2**$^{5+}$ (triplet):

Ru	4.19644256	14.14634114	22.90857710
N	5.54983239	14.55479200	21.31208998
N	5.62975313	12.57551494	23.02360102
N	3.55420755	12.97177742	24.56703595
N	2.74579021	13.26247803	21.58322468
N	5.09218180	15.62539388	24.18967595
N	-0.03482873	18.58085183	22.70984395
C	5.45036083	15.59923660	20.46938696
H	4.61562672	16.26866466	20.63428129
C	6.36087415	15.81904937	19.43997385
H	6.23386103	16.67660930	18.78929295
C	7.41577985	14.92505505	19.27407461
C	7.52498229	13.84229135	20.14326068
H	8.33824819	13.13549554	20.03192560
C	6.58408884	13.66906406	21.15886776
C	6.62881353	12.54720708	22.12254668
C	7.57580357	11.51915496	22.16427575
H	8.38938470	11.47248703	21.45099752
C	7.45208537	10.54177226	23.15288294
C	6.40617379	10.59282035	24.07560523
H	6.31623493	9.82965240	24.83892537
C	5.48931049	11.64426897	23.98471544
C	4.31947219	11.87396868	24.86044278
C	3.99045818	11.04177792	25.93108255
H	4.60433124	10.17734545	26.15287333
C	2.87329112	11.32658249	26.71258966
C	2.10334136	12.44554599	26.40509709
H	1.22554079	12.70917474	26.98385232
C	2.47635131	13.24158214	25.32647568
H	1.90589670	14.12045977	25.05370751
C	2.81072439	12.05848495	20.99534718
H	3.69023219	11.46411460	21.21485678
C	1.81341447	11.58154099	20.14706853
H	1.91543226	10.60064825	19.69696449
C	0.70471730	12.38812110	19.90098238
H	-0.09180490	12.05076913	19.24573802
C	0.62882064	13.64069270	20.50858243
H	-0.22496879	14.28451065	20.33031240
C	1.65797281	14.06753130	21.34895527
C	1.69848132	15.36524072	22.05084729
C	0.73600130	16.36828709	22.00880194
H	-0.15949030	16.27434903	21.40457418
C	0.93520988	17.55645787	22.75294517
C	2.10640060	17.74811042	23.52520337
H	2.20951461	18.66643715	24.09218007

C	3.07345132	16.74989093	23.55979273
C	2.86870839	15.55762416	22.82423317
C	4.33750607	16.76546494	24.32114676
C	4.78127368	17.82003222	25.12024541
H	4.17756694	18.71531170	25.21634792
C	5.99984880	17.71720234	25.78944169
H	6.35026875	18.53364621	26.41254817
C	6.75656798	16.55635213	25.64575901
H	7.71017337	16.43245343	26.14621003
C	6.26471037	15.53514074	24.83561351
H	6.82012486	14.61488256	24.69563470
N	3.01613672	23.64090380	21.12668812
N	0.42330769	24.46139910	24.33222704
C	3.91165449	24.29468917	20.36443423
H	4.05125168	25.34566159	20.59229530
C	4.62632920	23.68099739	19.34139035
H	5.33593651	24.25781562	18.75864528
C	4.40570659	22.32474452	19.09397522
H	4.94484915	21.81048297	18.30449182
C	3.48197141	21.63763067	19.87471799
H	3.29414924	20.58382894	19.70025957
C	2.78849381	22.30430700	20.89147185
C	1.79170168	21.69212777	21.78275118
C	1.36146319	20.36399125	21.77394032
H	1.74273062	19.64019171	21.05947679
C	0.39359856	19.94660042	22.70808824
C	-0.15403289	20.84374292	23.64555347
H	-0.88442107	20.47236083	24.35857314
C	0.26870106	22.17422264	23.64554786
C	1.24598781	22.60483729	22.71666266
C	-0.18067632	23.24415278	24.54884062
C	-1.13696512	23.09993452	25.56070287
H	-1.60479378	22.13479587	25.72182115
C	-1.48329959	24.18915741	26.35360470
H	-2.22436312	24.08025977	27.13933264
C	-0.86458681	25.41926438	26.12264323
H	-1.10262503	26.29623173	26.71437235
C	0.07897532	25.50770767	25.10483630
H	0.58423279	26.44277922	24.88990821
N	-5.96014722	19.09331804	21.35489412
N	-5.32273523	15.76680153	23.88822410
C	-6.98807329	19.70149335	20.74362375
H	-7.96689594	19.26250696	20.89920266
C	-6.82623718	20.83511485	19.94990545
H	-7.69068552	21.28775888	19.47766185
C	-5.54524702	21.35790154	19.78637894
H	-5.38020616	22.23968128	19.17574888
C	-4.47186497	20.73336814	20.41961535
H	-3.46698933	21.12444219	20.30826053
C	-4.69245264	19.59930616	21.20238868
C	-3.64644800	18.85077271	21.92615440
C	-2.28311853	19.12156054	21.93895827
H	-1.85687364	19.94584137	21.37827634
C	-1.41334076	18.28686620	22.68280495
C	-1.91191208	17.18336155	23.41760126
H	-1.21876354	16.58844980	24.00179310
C	-3.27395828	16.90460966	23.39459788
C	-4.14230765	17.73984954	22.65079015

C	-3.96501868	15.80288988	24.09242900
C	-3.34491379	14.84884585	24.90058145
H	-2.27257304	14.88658322	25.05492542
C	-4.10797359	13.85152702	25.50588942
H	-3.63174064	13.10732421	26.13592103
C	-5.48393109	13.82588696	25.28992472
H	-6.11708421	13.06928922	25.73918023
C	-6.04974391	14.80307583	24.47348323
H	-7.11589769	14.82007401	24.27838135
Ru	1.85761052	24.46794301	22.73423060
N	0.50887039	25.43286566	21.40006331
N	2.50966023	26.44703312	22.76198378
N	3.50901726	24.40870321	24.07833817
C	-0.50116053	24.85650771	20.72237252
H	-0.62657083	23.79212689	20.88119819
C	-1.33990405	25.56540138	19.86796098
H	-2.13754756	25.04695569	19.34758003
C	-1.12975840	26.93373045	19.70412601
C	-0.08737939	27.54110978	20.39906173
H	0.09440673	28.60336098	20.28679286
C	0.72535622	26.77956074	21.24256783
C	1.85390240	27.35496067	22.00858831
C	2.26921540	28.69029914	22.01015208
H	1.76171267	29.43884465	21.41340699
C	3.36145670	29.04938231	22.80263549
C	4.02274903	28.09180380	23.57444840
H	4.86885327	28.37833857	24.18762957
C	3.56811799	26.76986061	23.53475137
C	4.13135672	25.61699341	24.27310471
C	5.23348336	25.72503199	25.12496086
H	5.71487833	26.68516531	25.26789132
C	5.71191017	24.59855788	25.78867142
C	5.07268978	23.37649033	25.58581159
H	5.40746201	22.47143568	26.08040822
C	3.97987052	23.32685271	24.72593719
H	3.45212613	22.39968337	24.53709616
Ru	-6.03989508	17.34425107	22.61095733
N	-6.26126432	16.03596849	20.94263514
N	-8.12210410	16.90653491	22.56437571
N	-6.83985561	18.43381845	24.25992058
C	-5.25450303	15.62570827	20.14944957
H	-4.26873570	15.99987077	20.39522086
C	-5.45348228	14.76939422	19.07077863
H	-4.60662349	14.47043342	18.46378507
C	-6.74306359	14.31867020	18.79984432
C	-7.78969099	14.73878164	19.61719870
H	-8.80021236	14.39970602	19.42476080
C	-7.53403313	15.59821021	20.68637968
C	-8.58754401	16.08946640	21.60176107
C	-9.95109858	15.78547360	21.54515435
H	-10.35228780	15.13102189	20.78113988
C	-10.79751410	16.34712053	22.50239022
C	-10.29043746	17.19174552	23.49104059
H	-10.95344152	17.62455801	24.23013065
C	-8.91807924	17.45926452	23.49788608
C	-8.19109267	18.32071264	24.45630794
C	-8.81599782	18.98925406	25.50959297
H	-9.88488872	18.89082275	25.65445641

C	-8.06234761	19.78110167	26.37268726
C	-6.68960584	19.88806162	26.16344184
H	-6.06068211	20.49106740	26.80826776
C	-6.11731826	19.19965668	25.09796144
H	-5.05431434	19.25379919	24.89995364
H	-8.54277504	20.30310989	27.19327755
H	-11.85890819	16.12519560	22.47762970
H	-6.93579507	13.65082570	17.96705951
H	-1.76373429	27.52041313	19.04757029
H	3.69886494	30.08024600	22.81881429
H	6.56754787	24.67695840	26.45118218
H	8.14448613	15.06431116	18.48273216
H	8.17484063	9.73465344	23.20426325
H	2.61271416	10.68380586	27.54663248

Cartesian coordinates of DFT-optimized structures of **2**$^{6+}$ (doublet):

Ru	4.08590953	14.10613388	22.72191014
N	5.47421518	14.59983488	21.18029976
N	5.47063652	12.48080930	22.71333175
N	3.40097918	12.81593080	24.27356811
N	2.62639614	13.37195956	21.31582962
N	5.01361141	15.44601719	24.12932122
N	0.02212715	18.69228434	22.78538363
C	5.41968252	15.71891855	20.43477564
H	4.60527703	16.39892328	20.64895595
C	6.35161884	15.99947933	19.44001666
H	6.26106963	16.91600025	18.86849687
C	7.37983866	15.08974554	19.20641893
C	7.44152178	13.92996490	19.97521552
H	8.23302960	13.20880046	19.81174330
C	6.48115292	13.69803678	20.95983568
C	6.47697765	12.49615021	21.82087120
C	7.38844456	11.43697151	21.77817379
H	8.20621857	11.42590785	21.06830881
C	7.22314472	10.38243672	22.67722001
C	6.17154507	10.38984341	23.59487712
H	6.04987785	9.56830837	24.29005495
C	5.29149882	11.47600807	23.58982094
C	4.12423205	11.67074594	24.47640048
C	3.75696835	10.76415587	25.47121672
H	4.33773684	9.86251944	25.62259544
C	2.64407020	11.02277813	26.26801356
C	1.91740086	12.19092749	26.05251190
H	1.04484007	12.43706884	26.64654130
C	2.32756328	13.06124102	25.04742200
H	1.79068961	13.97977033	24.84815417
C	2.64990194	12.20721038	20.65180536
H	3.50120240	11.56352886	20.83989771
C	1.64347540	11.82824866	19.76475691
H	1.71083168	10.87514895	19.25283323
C	0.57178255	12.69370091	19.56193576
H	-0.23044293	12.43289855	18.87947672
C	0.53932867	13.90651902	20.24982348
H	-0.28674659	14.59384650	20.10759932
C	1.57475056	14.23372381	21.12477557
C	1.65437504	15.47742343	21.91639632
C	0.73507775	16.52050455	21.92849559

H	-0.15033765	16.51167116	21.30251615
C	0.97142663	17.63831259	22.76206686
C	2.12405080	17.72913534	23.57649853
H	2.24294275	18.59356590	24.22047253
C	3.05188419	16.69320980	23.55407057
C	2.81186659	15.56941063	22.72697553
C	4.29953423	16.60023749	24.33745780
C	4.76400072	17.57212532	25.22336063
H	4.19208059	18.47971654	25.37880899
C	5.96394068	17.37042010	25.90468671
H	6.33129267	18.12173654	26.59602853
C	6.67915067	16.19609117	25.68409446
H	7.61641396	15.99659177	26.19080581
C	6.16758894	15.25985708	24.78716961
H	6.69264612	14.33312542	24.58622947
N	3.32884602	23.63027681	21.43775560
N	0.17022716	24.65055547	24.05732062
C	4.34188412	24.24615504	20.81039602
H	4.43138331	25.31326292	20.97806785
C	5.23748270	23.56833894	19.98545680
H	6.03905887	24.11391274	19.50078499
C	5.07407313	22.19643306	19.80742098
H	5.75210013	21.63498451	19.17298853
C	4.02438974	21.54857506	20.45728732
H	3.88060970	20.48116507	20.33481771
C	3.15676934	22.27868461	21.26990428
C	2.01239106	21.71601938	22.01295540
C	1.59010930	20.39547402	22.01693291
H	2.08615499	19.63481845	21.42427995
C	0.45247348	20.03088068	22.78392580
C	-0.26116919	20.99300869	23.54576821
H	-1.10392244	20.66414027	24.14341263
C	0.15438984	22.31558933	23.53239738
C	1.29376070	22.68208992	22.76710516
C	-0.45782594	23.44612736	24.25730115
C	-1.58051116	23.35937229	25.08104349
H	-2.07000505	22.40360605	25.22959677
C	-2.06853963	24.50449757	25.70885779
H	-2.94085299	24.44436330	26.35143603
C	-1.42174281	25.72018996	25.49877400
H	-1.76554524	26.63611524	25.96571445
C	-0.30595806	25.74799770	24.66440700
H	0.22636424	26.67223196	24.47137563
N	-5.93176353	18.98844511	21.47327562
N	-5.17588319	15.80867770	24.16105355
C	-6.98293937	19.53170673	20.84200225
H	-7.94585532	19.06972207	21.02677724
C	-6.86219514	20.62974650	19.99204320
H	-7.74383868	21.03068611	19.50527252
C	-5.60043502	21.18429812	19.79277807
H	-5.46793475	22.03991216	19.13875970
C	-4.50260497	20.62606454	20.44704197
H	-3.51228096	21.04431073	20.30842092
C	-4.68240117	19.52610332	21.28531916
C	-3.60837763	18.84796929	22.03833543
C	-2.25229539	19.15334213	22.02004410
H	-1.85145811	19.94729899	21.39974866
C	-1.36053329	18.38352809	22.80434706

C	-1.81337934	17.30594689	23.60138918
H	-1.09495265	16.76269258	24.20514555
C	-3.16681816	16.98807959	23.60647172
C	-4.06093019	17.76222019	22.82710920
C	-3.82046443	15.89962930	24.36001192
C	-3.16740148	15.00811455	25.21077505
H	-2.09696337	15.08909825	25.36104609
C	-3.89669014	14.01560557	25.86512330
H	-3.39533463	13.31941406	26.52941649
C	-5.27048808	13.93306340	25.65311241
H	-5.87747995	13.17789693	26.13917139
C	-5.87014486	14.85064479	24.79198904
H	-6.93614174	14.82294654	24.59820767
Ru	1.86837723	24.52423742	22.74125818
N	0.79126194	25.37839983	21.10996364
N	2.50578091	26.55499073	22.70226941
N	3.25306114	24.66867280	24.35664556
C	-0.08279553	24.71441065	20.33123195
H	-0.23253962	23.66804191	20.56470988
C	-0.76300582	25.32363575	19.28118347
H	-1.45628895	24.74091894	18.68559951
C	-0.53214815	26.67267620	19.02390760
C	0.37191953	27.36602476	19.82499607
H	0.56729648	28.41569536	19.64285855
C	1.02700758	26.70578497	20.86504680
C	1.99885087	27.37106920	21.76021669
C	2.40668601	28.70718973	21.70292733
H	2.01544791	29.38494360	20.95413787
C	3.33917280	29.15775008	22.63859843
C	3.84981981	28.29166732	23.60679663
H	4.57362576	28.64889578	24.32890778
C	3.40529321	26.96599529	23.61472963
C	3.82396949	25.89919020	24.55051022
C	4.74347550	26.10353300	25.57997852
H	5.18693985	27.08115807	25.72386663
C	5.08862784	25.04835176	26.42107396
C	4.50288443	23.80204806	26.21379216
H	4.73907840	22.95046142	26.84145712
C	3.59150130	23.65352134	25.17282838
H	3.11333996	22.70231513	24.97692792
Ru	-5.94427524	17.30242220	22.81405270
N	-6.13907348	15.90822547	21.21310568
N	-8.01768356	16.79228607	22.80224343
N	-6.77982743	18.44031384	24.41115452
C	-5.12149936	15.49700458	20.43425059
H	-4.14815936	15.91634919	20.65449543
C	-5.29568618	14.58376842	19.39894261
H	-4.44160556	14.28657232	18.80142556
C	-6.56963855	14.07549770	19.15846674
C	-7.62671101	14.49701870	19.96164117
H	-8.62561260	14.11421257	19.79223169
C	-7.39651504	15.41497179	20.98662189
C	-8.46034250	15.91315026	21.88495345
C	-9.81212669	15.55792765	21.85489476
H	-10.19405878	14.85273620	21.12708257
C	-10.67111752	16.13346754	22.79229849
C	-10.18779972	17.04198693	23.73503279
H	-10.85971602	17.48346538	24.46071540

C	-8.82650310	17.35995589	23.71506767
C	-8.12511496	18.29347943	24.62219432
C	-8.76678137	18.99839316	25.64059223
H	-9.83145330	18.87460305	25.79599693
C	-8.03533221	19.86023335	26.45435476
C	-6.66773240	19.99925303	26.23169211
H	-6.05574986	20.65628368	26.83877303
C	-6.07776792	19.27319946	25.20163007
H	-5.01816627	19.35165983	24.99483766
H	-8.52894763	20.41118122	27.24767591
H	-11.72365308	15.87175203	22.78850884
H	-6.74250589	13.36221137	18.35977571
H	-1.04459440	27.18077124	18.21401939
H	3.67028184	30.19028941	22.61293942
H	5.80271212	25.20140401	27.22314527
H	8.12428114	15.27553961	18.43972830
H	7.91784295	9.54961529	22.66292698
H	2.35363168	10.32182852	27.04319378

Cartesian coordinates of DFT-optimized structures of **2**$^{6+}$ (quartet):

Ru	4.09889198	14.10540685	22.74658557
N	5.49503773	14.60138463	21.21425521
N	5.48516550	12.47988504	22.74277195
N	3.41320764	12.81557927	24.29851173
N	2.64423703	13.36771016	21.33486455
N	5.01421757	15.45077968	24.15728009
N	0.01338733	18.68546959	22.79268178
C	5.44331166	15.72324108	20.47281216
H	4.62816897	16.40257772	20.68680684
C	6.37891756	16.00653866	19.48229221
H	6.29105472	16.92501114	18.91350260
C	7.40751971	15.09695997	19.24941612
C	7.46606597	13.93446466	20.01439422
H	8.25804301	13.21367497	19.85149833
C	6.50198049	13.69959627	20.99466856
C	6.49362456	12.49556435	21.85261125
C	7.40334127	11.43493037	21.80951035
H	8.22244897	11.42396735	21.10116204
C	7.23441567	10.37878656	22.70601873
C	6.18113794	10.38620933	23.62175790
H	6.05678076	9.56358864	24.31518018
C	5.30285119	11.47379897	23.61699679
C	4.13444810	11.66932240	24.50184269
C	3.76457828	10.76312779	25.49602688
H	4.34378181	9.86057874	25.64798579
C	2.65118012	11.02352946	26.29155835
C	1.92679606	12.19304303	26.07564382
H	1.05405118	12.44048340	26.66885893
C	2.33950092	13.06299355	25.07126567
H	1.80453091	13.98254840	24.87135145
C	2.67293118	12.20370950	20.66958175
H	3.52563842	11.56235091	20.85937656
C	1.67031171	11.82261393	19.77934660
H	1.74176149	10.87025364	19.26660178
C	0.59686095	12.68552528	19.57467338
H	-0.20279633	12.42325493	18.88970195
C	0.55902345	13.89746617	20.26381948

H	-0.26861430	14.58262159	20.12024745
C	1.59078922	14.22725795	21.14228091
C	1.66379311	15.47100880	21.93493075
C	0.74372556	16.51627696	21.93810005
H	-0.13701023	16.50470713	21.30546605
C	0.96753995	17.63227674	22.77494074
C	2.11199504	17.72154985	23.59753513
H	2.22862963	18.58734098	24.24030911
C	3.04561493	16.68734774	23.57863599
C	2.81298899	15.56528956	22.75178722
C	4.29243378	16.60082085	24.36402128
C	4.74984393	17.57483312	25.25151556
H	4.17197114	18.47878804	25.40614496
C	5.94955913	17.38000483	25.93517489
H	6.31112356	18.13330277	26.62745509
C	6.67212843	16.20991106	25.71593109
H	7.60946835	16.01562368	26.22452874
C	6.16774245	15.27108458	24.81770420
H	6.69861418	14.34744421	24.61775674
N	3.29533040	23.63924737	21.38120751
N	0.20835216	24.65079402	24.09318016
C	4.29350271	24.25462066	20.73076707
H	4.39428097	25.31985541	20.90360435
C	5.16179514	23.57783952	19.87588229
H	5.95269244	24.12250685	19.37310357
C	4.98511008	22.20868245	19.69274859
H	5.64181165	21.64805276	19.03558115
C	3.94978249	21.56143554	20.36676837
H	3.79616883	20.49595345	20.24012694
C	3.10955262	22.28972282	21.20861582
C	1.98180932	21.72585705	21.97717688
C	1.55033698	20.40202206	21.98090202
H	2.02651651	19.64403370	21.36874592
C	0.44695957	20.03585728	22.78485403
C	-0.23475495	20.98639450	23.57827038
H	-1.05956809	20.65491486	24.19935264
C	0.17903087	22.31584587	23.55575278
C	1.28883792	22.67786772	22.75896398
C	-0.41336986	23.44409415	24.30180353
C	-1.51398081	23.35255321	25.15369021
H	-1.99909162	22.39574126	25.30919683
C	-1.98604048	24.49435870	25.80043562
H	-2.84158904	24.42995248	26.46475299
C	-1.34586964	25.71145160	25.58090130
H	-1.67790606	26.62455975	26.06153680
C	-0.25177656	25.74462345	24.71801768
H	0.27473552	26.67033925	24.51645158
N	-5.94276946	18.98852570	21.46284850
N	-5.19650332	15.79800981	24.14138292
C	-6.99129301	19.53751556	20.83221760
H	-7.95602103	19.07829194	21.01456996
C	-6.86597384	20.63782328	19.98586416
H	-7.74569995	21.04348627	19.49953460
C	-5.60190782	21.18819387	19.78963258
H	-5.46559171	22.04534364	19.13836393
C	-4.50673318	20.62377987	20.44306324
H	-3.51456990	21.03825176	20.30625584
C	-4.69078993	19.52193764	21.27813437

C	-3.61918685	18.83771124	22.02928665
C	-2.26049140	19.14319072	22.01665107
H	-1.86005162	19.94233308	21.40265189
C	-1.37186771	18.37724644	22.80476750
C	-1.83056261	17.30200463	23.59894697
H	-1.11588035	16.75777816	24.20642272
C	-3.18494025	16.97869617	23.59292716
C	-4.07343705	17.75186498	22.81177597
C	-3.84086232	15.88767953	24.34120273
C	-3.18964820	14.99169195	25.18890886
H	-2.11913888	15.07091879	25.33961674
C	-3.92017735	13.99731721	25.83894617
H	-3.41968028	13.29793915	26.50059924
C	-5.29403536	13.91665366	25.62617485
H	-5.90195519	13.16010657	26.10891976
C	-5.89204544	14.83814128	24.76829502
H	-6.95796839	14.81218842	24.57365130
Ru	1.87339907	24.53195346	22.73160027
N	0.76462228	25.41622166	21.13973806
N	2.52263554	26.56680810	22.69534835
N	3.29857841	24.66413823	24.31032037
C	-0.13066450	24.75848016	20.38005718
H	-0.27776434	23.71103016	20.61029193
C	-0.83474591	25.37773280	19.35196183
H	-1.54492066	24.80155964	18.77020267
C	-0.60561969	26.72771984	19.09841597
C	0.32076861	27.41325791	19.88064001
H	0.51513098	28.46341577	19.70045214
C	0.99944153	26.74379254	20.89909042
C	1.99608420	27.39541569	21.77554219
C	2.40782864	28.73030668	21.72190447
H	2.00008774	29.41732412	20.99069544
C	3.36568158	29.16716478	22.63811815
C	3.89675688	28.28863694	23.58363999
H	4.63980839	28.63441876	24.29156199
C	3.44670238	26.96505684	23.58839275
C	3.88353079	25.88751798	24.50194964
C	4.83103582	26.07284072	25.50892081
H	5.28734117	27.04480195	25.65082731
C	5.18750385	25.00540035	26.32973680
C	4.58511998	23.76661218	26.12528282
H	4.82918483	22.90654040	26.73806457
C	3.64650834	23.63631260	25.10634583
H	3.15492723	22.69155944	24.91256372
Ru	-5.96349310	17.29736401	22.79814818
N	-6.15428770	15.91034091	21.19027343
N	-8.03760682	16.78558664	22.77730013
N	-6.80589582	18.42885175	24.39484162
C	-5.13369452	15.50466342	20.41251284
H	-4.16185344	15.92494874	20.63770041
C	-5.30356997	14.59569354	19.37274299
H	-4.44741826	14.30252844	18.77620090
C	-6.57599926	14.08623910	19.12663175
C	-7.63609438	14.50236921	19.92864808
H	-8.63387623	14.11862866	19.75474763
C	-7.41025328	15.41619821	20.95826201
C	-8.47703969	15.90943914	21.85573927
C	-9.82831861	15.55273425	21.82098233

H	-10.20754311	14.84976265	21.08961402
C	-10.69033390	16.12390711	22.75829758
C	-10.21042136	17.02965869	23.70544183
H	-10.88460868	17.46793223	24.43098427
C	-8.84944272	17.34916014	23.69007945
C	-8.15137612	18.28013797	24.60234832
C	-8.79542653	18.98113495	25.62194064
H	-9.86032568	18.85619535	25.77489525
C	-8.06588330	19.84050986	26.44005586
C	-6.69788164	19.98128937	26.22059621
H	-6.08762189	20.63662305	26.83123083
C	-6.10539976	19.25928697	25.18912657
H	-5.04544306	19.33869573	24.98410965
H	-8.56130317	20.38838029	27.23438478
H	-11.74255285	15.86100063	22.75094689
H	-6.74538689	13.37615149	18.32434946
H	-1.13674261	27.24282913	18.30521182
H	3.70027083	30.19860395	22.61512180
H	5.92341788	25.14340036	27.11456280
H	8.15484938	15.28509709	18.48611175
H	7.92767067	9.54477295	22.69132645
H	2.35864429	10.32292212	27.06626056
